# Role of NAD^+^ in regulating cellular and metabolic signaling pathways

**DOI:** 10.1016/j.molmet.2021.101195

**Published:** 2021-02-17

**Authors:** Sara Amjad, Sabah Nisar, Ajaz A. Bhat, Ab Rauf Shah, Michael P. Frenneaux, Khalid Fakhro, Mohammad Haris, Ravinder Reddy, Zoltan Patay, Joseph Baur, Puneet Bagga

**Affiliations:** 1 Shibli National College, Azamgarh, Uttar Pradesh, India; 2 Functional and Molecular Imaging Laboratory, Cancer Research Department, Sidra Medicine, Doha, Qatar; 3 University of Nebraska-Lincoln, Lincoln, NE, USA; 4 Academic Health System, Hamad Medical Corporation, Doha, Qatar; 5 Department of Human Genetics, Sidra Medicine, Doha, Qatar; 6 Department of Genetic Medicine, Weill Cornell Medical College, Doha, Qatar; 7 Laboratory Animal Research Center, Qatar University, Doha, Qatar; 8 Department of Radiology, University of Pennsylvania, Philadelphia, PA, USA; 9 Department of Diagnostic Imaging, St. Jude Children's Research Hospital, Memphis, TN, USA; 10 Department of Physiology and Institute for Diabetes, Obesity, and Metabolism, University of Pennsylvania, Philadelphia, PA, USA

**Keywords:** NAD^+^, Aging, Cancer, Metabolism, Neurodegeneration, Sirtuins

## Abstract

**Background:**

Nicotinamide adenine dinucleotide (NAD^+^), a critical coenzyme present in every living cell, is involved in a myriad of metabolic processes associated with cellular bioenergetics. For this reason, NAD^+^ is often studied in the context of aging, cancer, and neurodegenerative and metabolic disorders.

**Scope of review:**

Cellular NAD^+^ depletion is associated with compromised adaptive cellular stress responses, impaired neuronal plasticity, impaired DNA repair, and cellular senescence. Increasing evidence has shown the efficacy of boosting NAD^+^ levels using NAD^+^ precursors in various diseases. This review provides a comprehensive understanding into the role of NAD^+^ in aging and other pathologies and discusses potential therapeutic targets.

**Major conclusions:**

An alteration in the NAD^+^/NADH ratio or the NAD^+^ pool size can lead to derailment of the biological system and contribute to various neurodegenerative disorders, aging, and tumorigenesis. Due to the varied distribution of NAD^+^/NADH in different locations within cells, the direct role of impaired NAD^+^-dependent processes in humans remains unestablished. In this regard, longitudinal studies are needed to quantify NAD^+^ and its related metabolites. Future research should focus on measuring the fluxes through pathways associated with NAD^+^ synthesis and degradation.

## Highlights

• NAD^+^ regulates energy metabolism, DNA damage repair, gene expression, and stress response.
• NAD^+^ deterioration contributes to the progression of multiple metabolic disorders, cancers, and neurodegenerative diseases.
• Nicotinamide mononucleotide and nicotinamide riboside raise NAD^+^ levels in different tissues in preclinical models.
• Imaging studies on genetic models can illustrate the pathways of NAD^+^metabolism and their downstream functional effects.
• Human clinical trials to determine benefits of restoration of NAD^+^ by using NAD precursors are in progress.

## Graphical abstract

**Figure:**
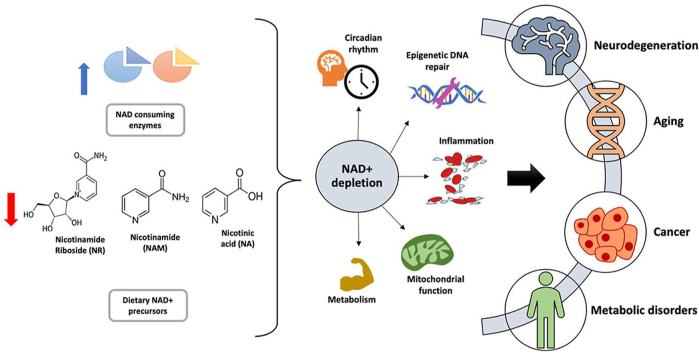

## 1 Introduction

Since the discovery of nicotinamide adenine dinucleotide (NAD), researchers have progressively learned more about its roles in cellular function. NAD^+^ has emerged as a critical modulator of cell signaling and survival pathways [1]. Cellular NAD exists in two forms, oxidized (NAD^+^) and reduced (NADH) [2]. NAD^+^ and another essential intracellular coenzyme flavin adenine dinucleotide (FAD^+^) play essential roles in cellular oxidation-reduction (redox) reactions and are responsible for accepting high-energy electrons and carrying them to the electron transport chain (ETC) to synthesize adenosine triphosphate (ATP) [3]. Regulation and maintaining a proper balance of the NAD^+^/NADH and FADH_2_/FAD ratio is critical for normal cell function and viability [4]. NAD^+^ acts as a cofactor for enzymes involved in cellular energy metabolism and various metabolic pathways such as glycolysis, fatty acid oxidation, and the citric acid cycle [5]. Both NAD^+^ and NADH play important roles as coenzymes in redox reactions, and an imbalance in their ratio can impair flux through these pathways' reactions, resulting in dysregulated cellular metabolism.

However, ATP generated via glycolytic reactions is critical for NAD + regeneration from NADH [6]. The crucial role of NAD^+^ in different biological functions such as aging, metabolism, mitochondrial function, immunological pathways, oxidative stress, gene expression, and apoptosis has been extensively investigated [7]. Many studies have found that altered NAD^+^ levels play an important role in metabolic disorders, neurodegenerative disorders, and tumorigenesis [8,9]. In this review, we discuss the importance of cellular NAD^+^ in aging, neurodegeneration, metabolic disorders, and cancer.

## 2 NAD^+^ biosynthesis pathways

**Figure 1:**
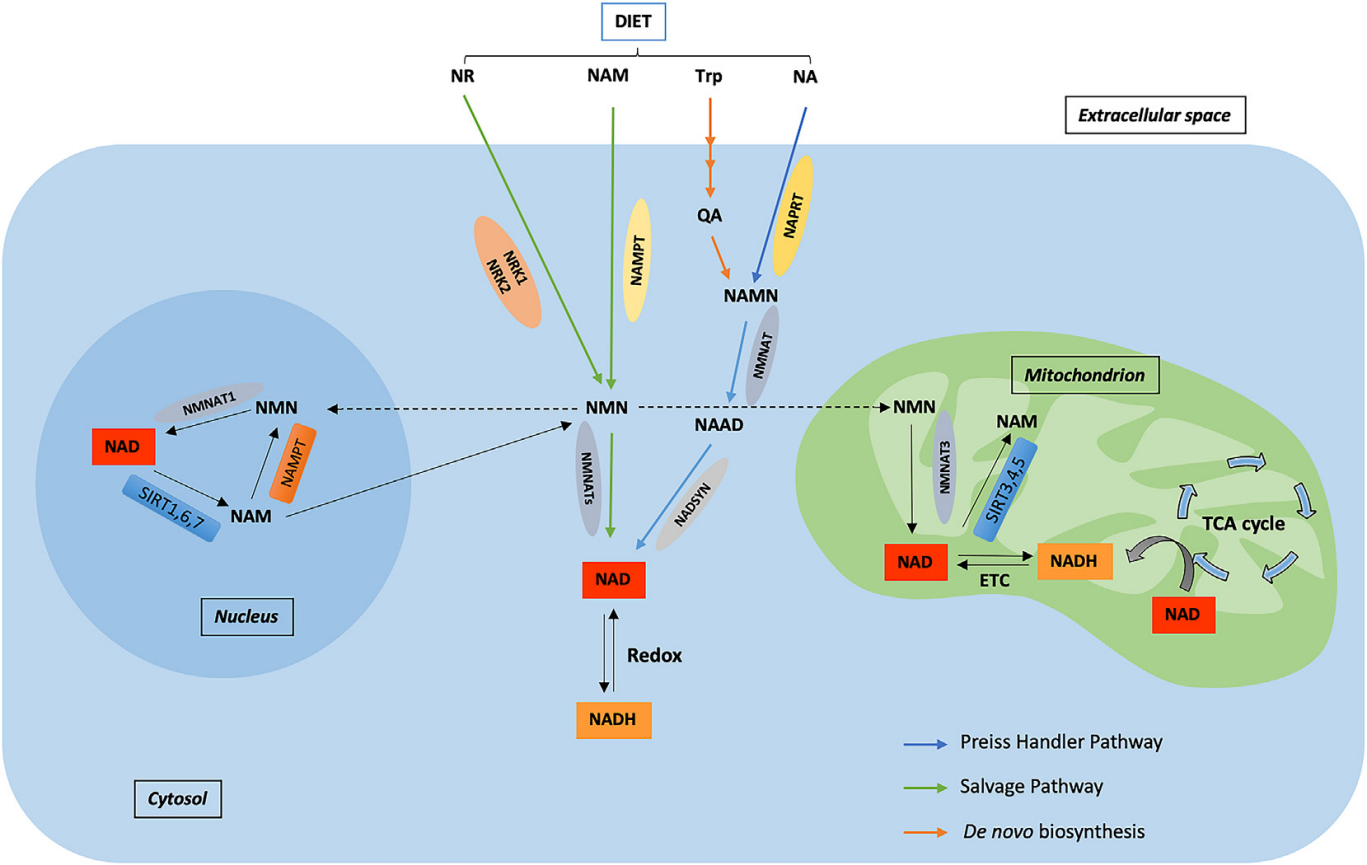
**Representation of NAD**^**+**^**biosynthesis pathways.** Biosynthesis pathways and cellular metabolism of NAD^+^. NAD precursors such as NR, NA, NAM, and Trp provided by diet can be converted into NAD via three pathways. In the Preiss-Handler pathway, NA is converted into NAMN by NAPRT, NAMN is converted into NAAD by NAD by NMNATs, and NAAD is converted into NAD by NADSYN. In the de novo synthesis pathway, Trp is converted into QA in a series of steps, which is then converted into NAD by forming NAMN and NAAD. In contrast, in the salvage pathway, NR and NAM provided by diet are converted into NAD by forming NMN by enzymes NAMPT and NRK [2]. The equilibrium in each subcellular compartment such as the nucleus and mitochondria is determined by NAD/NADH redox ratios. ETC is a significant contributor to the conversion of NADH into NAD. Additionally, NAD-consuming enzymes such as PARPs and sirtuins catalyze NAM production in subcellular compartments, which can be used for NAD synthesis via the salvage pathway.

The intracellular concentration of NAD^+^ is a balance between NAD^+^ consumption and synthesis. The biosynthetic pathways of NAD^+^ play an important role in maintaining NAD^+^ pools, which are not only required for fueling redox metabolism but also to support NAD^+^-dependent signaling pathways. As NAD^+^-dependent signaling pathways involve the degradation of NAD^+^, cells require continuous replenishment of NAD^+^ that can be accomplished by an efficient NAD^+^ biosynthesis [10]. The three canonical pathways for the synthesis of NAD^+^ in mammalian cells are the Preiss-Handler pathway, de novo biosynthesis pathway, and NAD^+^ salvage pathway (Figure 1) [11,12]. The formation of nicotinamide (NAM) or nicotinic acid (NA) adenine dinucleotides from the corresponding mononucleotides and ATP constitutes a critical step for NAD generation [13]. The three isoforms of nicotinamide mononucleotide adenylyl transferase (NMNAT1, 2, and 3) that catalyze this reaction have different tissue and subcellular distributions [13]. NMNAT-1 is highly expressed in the skeletal muscle, heart, kidney, liver, kidney, and pancreas [14,15]. In contrast, NMNAT-2 is highly expressed in the brain and NMNAT-3 is highly expressed in the erythrocytes, lungs, and spleen [[16], [17], [18]]. NMNAT-3 and in some reports a minority of nicotinamide phosphoribosyltransferase (NAMPT) [19], which lies upstream of NMNAT-3, were found to be mitochondrial, yet their localization status remains controversial. In contrast, all of the other enzymatic activities associated with NAD^+^ biosynthesis occur in the cytosol and/or nucleus [20]. To identify the factors that contribute to the reduction of NAD^+^ levels associated with aging and its related pathologies, including cancer, it is important to review the NAD^+^ biosynthesis pathways.

### 2.1 Preiss-Handler pathway

NA, which can be obtained from foods such as meat, redfish, and nuts and is also produced by the microbiome, is converted into NAD^+^ through the Preiss-Handler pathway. In this pathway, NA mononucleotide (NAMN) forms from the reaction of NA with phosphoribosyl pyrophosphate (PRPP), a pentose phosphate that is catalyzed by NA phosphoribosyltransferase (NAPRT) [21]. NMNATs then convert NAMN into NA adenine dinucleotide (NAAD), and then NAAD is converted into NAD^+^ by glutamine-dependent NAD synthase.

### 2.2 De novo biosynthesis pathway

This is the longest of the NAD^+^ synthesis pathways and is active mainly in the liver and kidneys. The amino acid tryptophan (Trp) is catabolized through the kynurenine (KYN) pathway (comprising 9 steps) to generate quinolinic acid (QA) [22]. In the first rate-limiting step, Trp is oxidized to form *N*-formylkynurenine (NFK) by either indoleamine-2,3-dioxygenase (IDO) or tryptophan-2,3-dioxygenase (TDO) [23]. NFK then transforms into KYN, which results in either 3-hydroxykynurenine or anthranilic acid forming 3-hydroxyanthranilic acid (3-HAA), which is finally converted into unstable α-amino-β-carboxymuconate-ε-semialdehyde (ACMS). The spontaneous cyclization of ACMS forms QA. The second rate-limiting step is the formation of NAMN from QA, which is catalyzed by quinolinate phosphoribosyltransferase. NAMN is then converted into NAD^+^ through the Preiss-Handler pathway [24,25]. However, ACMS may exit the NAD^+^ formation pathway if decarboxylated by ACMS decarboxylase, and inhibitors of this enzyme enhance NAD^+^ synthesis [26]. QA generated by these steps is then converted into NAD^+^, and conditions that lead to the accumulation of QA can cause neurotoxicity [2].

### 2.3 Salvage pathway

This is the major pathway for NAD^+^ biosynthesis in most tissues in mammals. Enzymatic activities of NAD-consuming enzymes produce NAM as a by-product. NAM can also be obtained from the diet. In the salvage pathway, the formation of nicotinamide mononucleotide (NMN) from NAM is catalyzed by NAMPT. NAM can also be methylated by nicotinamide N-methyltransferase (NNMT) to form 1-methyl-nicotinamide, which is further metabolized and excreted [27]. NMN also forms by phosphorylation of nicotinamide riboside (NR) by nicotinamide riboside kinase 1–2 (NRK 1–2) and then converted into NAD^+^ by NMNATs [2]. The cellular level of NAD^+^ can be increased either by stimulation or activation of enzymes involved in NAD^+^ biosynthesis such as NAD^+^ precursors NMN, NR, and NAM [12] or by inhibition of enzymes that consume or degrade NAD^+^ such as CD38, poly-ADP-ribose-polymerases (PARPs), and sterile alpha and toll-interleukin receptor-containing motif (SARM1) [11]. Flavonoids such as luteolin, luteolinidin, apigenin, quercetin, and kuromanin and a highly potent thiazoloquin(az)olin(on)e 78c are reported to be effective CD38 inhibitors that can boost NAD^+^ levels and have beneficial effects in various human diseases [[28], [29], [30], [31]]. PARP inhibitors such as olaparib, niraparib, rucaparib, talazoparib, veliparib, and PJ34 play important roles in preserving and/or boosting NAD^+^ levels in different pathologies [[32], [33], [34], [35], [36], [37], [38]].

## 3 Roles of NAD^+^ and sirtuins in cellular maintenance

**Figure 2:**
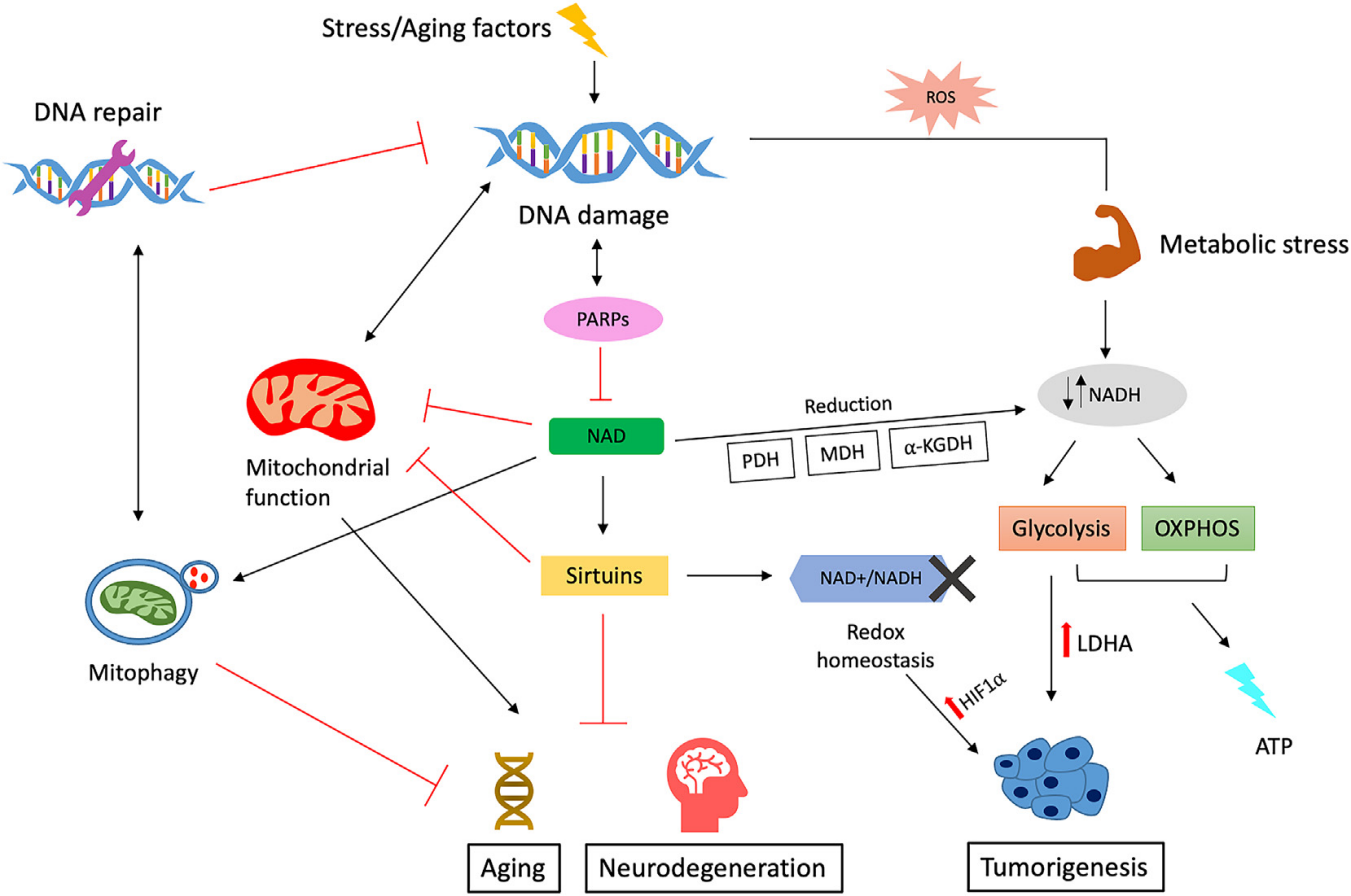
**Role of NAD in aging, neurodegeneration, and cancer.** DNA damage caused by stress or aging activates PARP. PARP activation leads to reduced levels of cytosolic NAD and mitochondrial dysfunction, which contribute to aging and neurodegeneration. Any disturbance in levels of NAD^+^/NADH (redox homeostasis) can upregulate oncogenic signaling pathways, leading to tumorigenesis.

Reduced levels of NAD^+^ coupled with a shift toward NADH are considered a hallmark of aging, although the underlying causes remain unclear [39]. Preclinical studies in aging models show that increasing NAD^+^ levels reduces age-related immune and metabolic changes and could potentially be used as a therapeutic strategy for treating aging-associated pathologies [12,[40], [41], [42]]. The role of NAD^+^ in various diseases has been evaluated using genetically modified mice or by boosting or replenishing NAD^+^ levels by administering precursors of NAD^+^ biosynthesis [43,44]. One crucial role of NAD^+^ in cellular maintenance is related to sirtuins. Sirtuins are NAD^+^-dependent deacylases and ADP ribosyltransferases that consume NAD^+^ and are crucial to maintaining and regulating cellular homeostasis. They may be affected by age-dependent declines in NAD^+^ levels [41,45]. The loss of sirtuins can lead to mitochondrial dysfunction, which can cause redox imbalance, leading to damaged cell proteins, lipids, and DNA. Damage to DNA can cause chromosomal aberrations and gene mutations, leading to the development of several chronic diseases, including cancer [46]. Reduced levels of sirtuins also lead to age-related diseases, as members of the sirtuin family such as sirtuin 1 (SIRT1) delay aging processes by catalyzing histone deacetylation and regulating transcription factors [47]. Nutritional and environmental factors significantly affect intracellular NAD^+^ levels. Declining levels of cellular NAD^+^ can impair sirtuin activities and alter the epigenetic chromatin structure [48] and mitochondrial metabolism, leading to increased oxidative stress and decreased ATP production. This increase in oxidative stress promotes inflammation, aggravating cellular injury [49] (Figure 2). Aging is characterized by a continued shift in metabolic activity, which increases from childhood to adulthood and declines afterward [50]. Although age-related metabolic activity changes correlate with cellular NAD^+^ levels [51], the exact mechanisms underlying decreased NAD^+^ levels remain unclear [39]. The expression and activity of NADase CD38 have also been associated with an age-related decline in NAD^+^ and may serve as potential therapeutic targets for age-related diseases [51]. Researchers are focusing on unraveling the molecular mechanisms involved in the physiological decline of NAD^+^ levels. Measuring changes in NAD^+^ levels in vivo may provide information about the altered redox potential associated with age-related diseases and can subsequently be linked with altered cellular metabolism (Figure 2). The decline in NAD^+^ with aging and in progeroid states (as reported in Werner and Cockayne syndromes) [52,53] can result in the development of various metabolic abnormalities [51]. As nuclear NAD + -dependent enzymes regulate mitochondrial function, it is suggested that an impairment in the oxidative phosphorylation (OXPHOS) mechanism during aging might be accelerated by the depletion of nuclear NAD^+^ [54]. Thus, to understand the molecular mechanisms underlying the aging process, it is crucial to study the effects of decreased cellular NAD^+^ levels and understand how different factors such as oxidative stress, inflammation, and DNA damage impact the cellular metabolism of NAD^+^ during aging. Many studies show a relationship between age-related illness, its comorbidities, and metabolic changes.

## 4 NAD^+^ in aging

**Figure 3:**
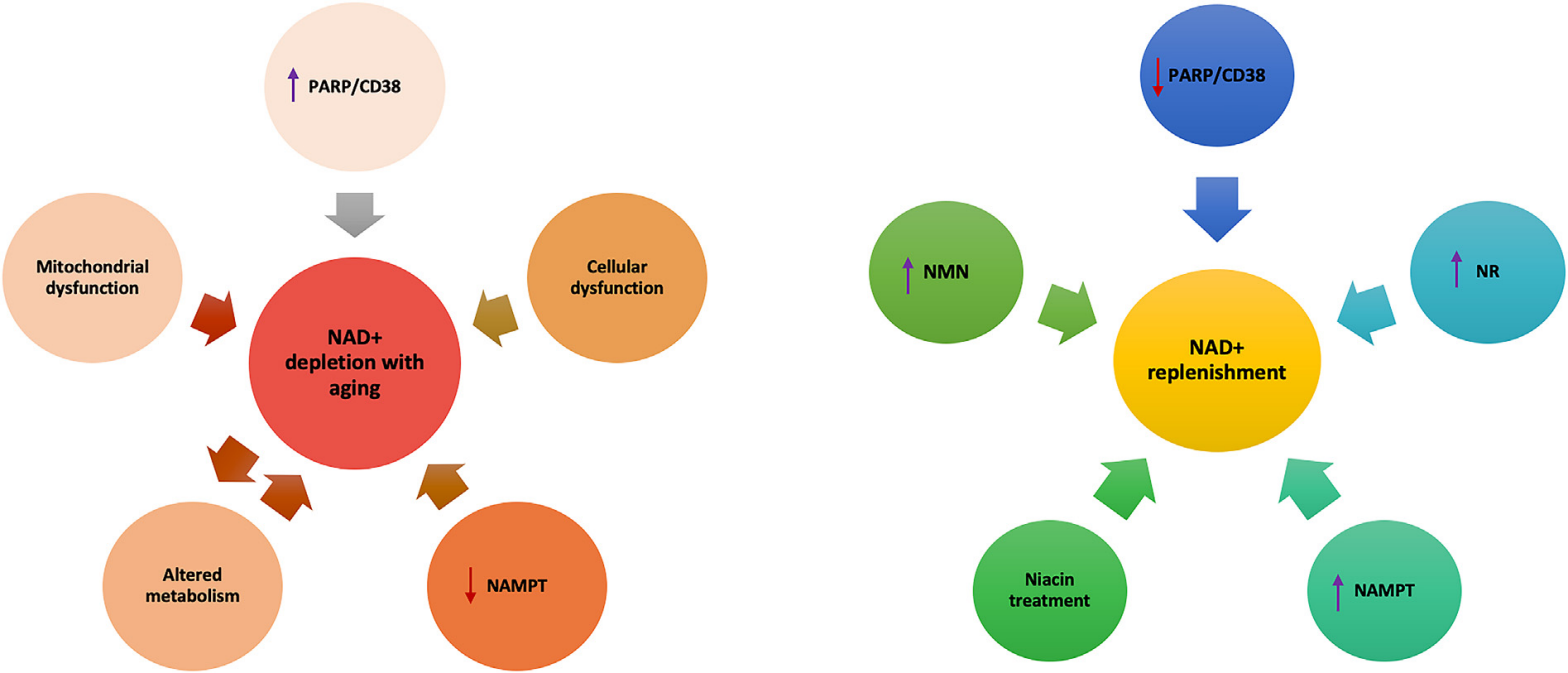
NAD^+^ depletion with increasing age is caused by several factors such as inefficient metabolism or protein consumption, increased activity of NAD^+^-consuming enzymes CD38/PARP, mitochondrial dysfunction, DNA damage, cellular dysfunction, and NAMPT depletion. NAD^+^ can be restored by manipulating enzymes in NAD^+^ synthesis pathways. The NAD^+^ concentration can be replenished by increasing the activity of NAMPT, niacin, NMN, and NR. Moreover, in many cases, inhibition of PARP/CD38 also helps restore NAD^+^.

The systemic decline in NAD^+^ has been associated with many hallmarks of aging. In addition to age-related mitochondrial content changes such as a change in the volume, integrity, and functionality of mitochondrial DNA due to increased ROS accumulation, other mechanisms also contribute to age-related declines in NAD^+^ [39,55]. The most recognized mechanisms include an increase in NAD^+^-consuming enzymes such as poly-ADP-ribose-polymerase 1 (PARP1) and SIRT1 [56,57] due to inflammation or DNA damage. PARP1 activation allows the recruitment of DNA repair proteins to repair damaged DNA. Although PARP1 activation is crucial for genomic maintenance, hyperactivation of PARP1 can cause a reduction in NAD^+^ levels [58]. NAD^+^ reduction leads to reduced SIRT1 activity, increasing proliferator-activated receptor gamma coactivator 1-alpha (PGC)-1α acetylation and decreasing transcriptional factor A mitochondria (TFAM) levels [59]. This decline in NAD^+^ levels can lead to cellular dysfunction and DNA damage and aggravate age-related pathologies. Changes in the mitochondrial content of cells with age can also compromise mitochondrial function and contribute to age-related NAD^+^ decline (Figure 3). Possible explanations for the age-related decline in NAD^+^ levels include increased expression of CD38 protein, dysregulation of circadian rhythms that reduce the expression of NAMPT, and high levels of PARP activity due to DNA damage, inflammation, or metabolic stress [[60], [61], [62]]. Studies show that blocking PARP activity can help recover NAD^+^ levels and mitochondrial function [54,62]. Pharmacological inhibition or deletion of the *PARP1* gene enhances mitochondrial content and oxidative metabolism in mice [33,63].

Supplementation of NAD^+^ precursor (NR) increases NAD^+^ levels, enhances oxidative metabolism, and protects against metabolic abnormalities, which SIRT1 and SIRT3 [64] may in part mediate. NR treatment enhances SIRT1 activity and leads to a higher *SOD2* expression, which results in the deacetylation and activation of FOXO1 [64]. NR treatment was also found to deacetylate PGC-1α in the muscle, liver, and brown adipose tissue, stimulate sirtuin activity, and enhance mitochondrial gene expression in NR-fed mice [64]. Another study found that treatment with NR rejuvenate muscle stem cells and delays senescence in neural and melanocyte stem cells in aged mice, thus increasing the mouse life span [41]. Cellular senescence associated with tissue decline alters adult stem cell functioning such as intestinal stem cells (ISCs) during aging. Supplementation of NR rejuvenates ISCs in aged mice and helps rescue repair defects in the aging gut [65].

NMN administration effectively mitigates age-associated physiological decline by suppressing age-associated weight gain and gene expression changes, enhancing energy and mitochondrial oxidative metabolism and improving insulin sensitivity and plasma lipid profiles in chow-fed wild-type C57BL/6N mice [40]. Studies in aging models show that NMN administration enhances energy metabolism and mitochondrial oxidative metabolism [40,66]. Interestingly, a study showed that NAD^+^ repletion restores microvasculature and capillaries' number and density in old mice [67]. Dietary treatment with NR has also been shown to improve cognitive function and synaptic plasticity in Alzheimer's disease (AD) by promoting the degradation of β-secretase through PGC-1α [68]. Augmentation of NAD^+^ levels by supplementing NR also improves cardiovascular and other physiological functions associated with aging, and NR is well tolerated in middle- and older-aged individuals [69]. Increasing intracellular NAD^+^ levels by administering NR/NMN delays memory loss, normalizes neuromuscular function, and extends life spans in ataxia-telangiectasia-deficient mice [70]. In some DNA repair disorders, the neurological phenotype is due to mitochondrial alterations such as in xeroderma pigmentosum [71]. Mitochondrial alterations are a consequence of reduced NAD^+^ levels due to the hyperactivation of PARP1, and these mitochondrial abnormalities can be rescued by inhibiting PARP1 or supplementation with NAD^+^ precursors [71]. Augmentation of NAD^+^ levels by administering NR rescues the mitochondrial phenotype in Xpa^−/−^/Csa^−/^ (CX) mice [71]. Aging is also associated with decreased levels of retinal NAD^+^, leading to eye conditions such as glaucoma that can damage the optic nerve. Oral administration of NA, an NAD^+^ precursor, protects mice against glaucoma development by rendering retinal ganglion cells more resistant to intraocular pressure [72]. All of these studies show that NAD^+^ and its associated metabolites are implicated in aging and its related pathologies.

## 5 Role of NAD^+^ in neurodegenerative disorders

**Figure 4:**
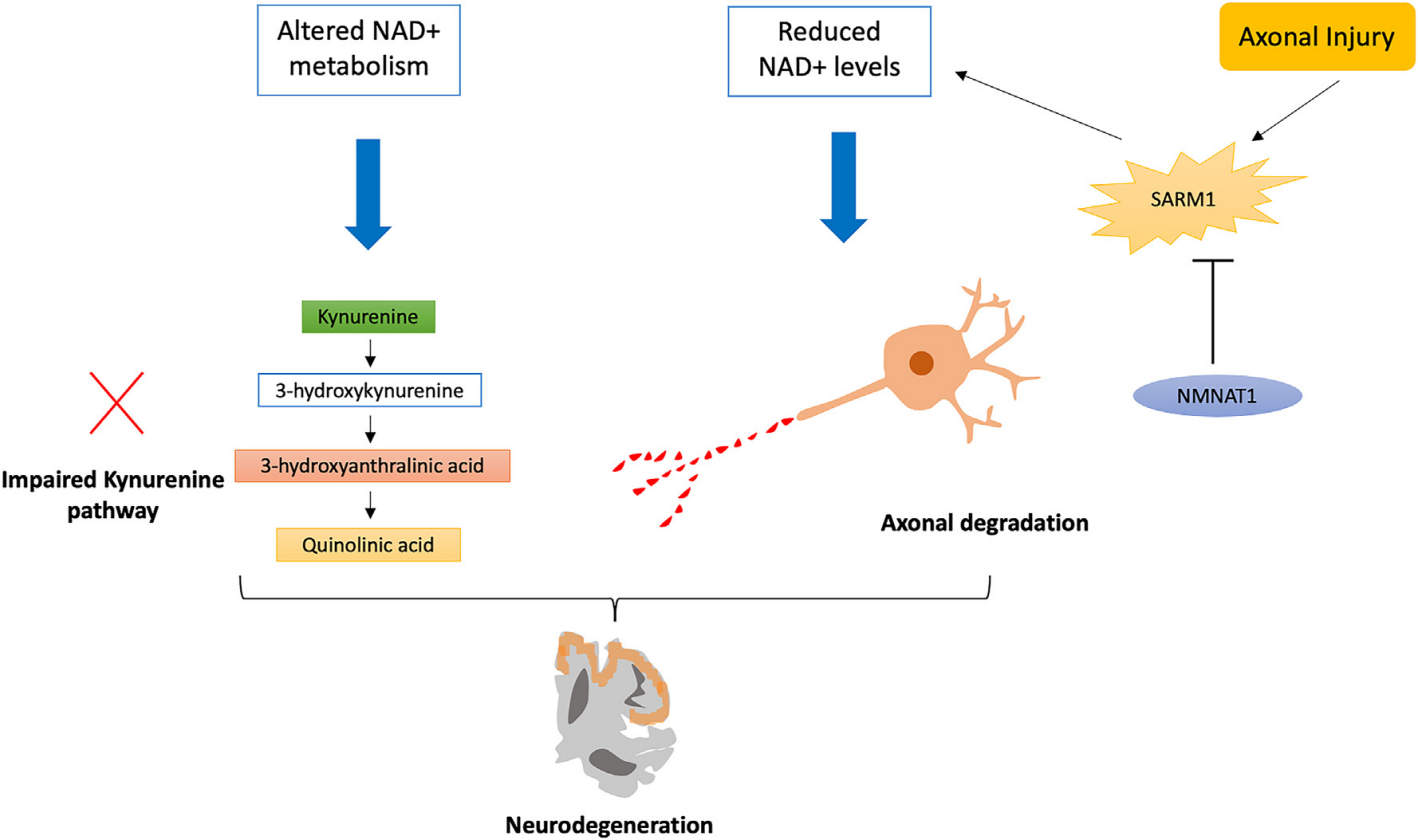
Altered NAD metabolism is associated with neurodegeneration. Axonal injury leads to the activation of SARM1, which reduces NAD + levels and leads to axonal degeneration. The overexpression of NMNAT1 inhibits SARM1 and protects injured axons. NAD levels are associated with axonal degradation, and impairment in the KYN pathway causes fluctuations in KYN pathway metabolite levels, which impairs the neurotransmission process and leads to neurodegeneration and the development of neurological disorders [201].

Mitochondrial dysfunction in neurons is a significant contributor to age-related neurodegenerative disorders such as AD and Parkinson's disease (PD) [73]. Aging contributes to the progression of neurodegenerative disorders, as it accelerates the production and accumulation of reactive oxygen species (ROS), triggering various cellular processes that contribute to DNA damage and impairment in mitochondrial functioning [74]. Several studies have documented NAD + - and NAD-dependent sirtuins' neuroprotective role in many neurodegenerative disorders [75,76]. Being the only de novo NAD^+^ synthesis pathway in mammals, the KYN pathway modulates neuronal functions and acts as a double-edged sword as it generates both neuroprotective metabolites and neurotoxic intermediates [77] (Figure 4). The maintenance of KYN pathway metabolites is determined by enzymes localized in the astrocytes and microglia in the brain, and any fluctuations in levels of these metabolites can impair neurotransmitter systems, which can lead to various neurological disorders [78]. For NAD^+^ depletion associated with aging-related neurodegenerative disorders, various NAD^+^ augmentation strategies such as supplementation/treatment with NAD^+^ precursors, PARP inhibitors (PARPi), or sirtuin activators can help restore mitochondrial function and enhance neuronal function, which can improve cognitive function [77]. Preclinical studies suggest that PARP1 inhibition helps treat AD, PD, and Huntington's disease (HD) [79,80]. Axonal degeneration is a hallmark of many neurological diseases, including neurodegenerative disorders, and delaying axonal degeneration can increase survival and decrease disease progression [81]. Activation of SARM1 is found to promote axonal degeneration by initiating a local destruction program that depletes NAD^+^ [82]. In contrast, the overexpression of NMNAT1 is found to inhibit axonal degradation by blocking SARM1-mediated NAD^+^ depletion [83]. Interestingly, elevated levels of NAD^+^ in CD38- and PARP1-deficient mice are found to have no effect on axonal degeneration and do not provide axonal protection, suggesting that the loss or deletion of these NAD^+^-consuming enzymes has no effect on NMNAT1-mediated axonal protection [81].

### 5.1 Alzheimer's disease

Alzheimer's disease (AD) is a progressive disorder characterized by impaired cognition and memory processes caused by neuronal degeneration. AD is the most common form of dementia. It worsens over time and affects the brain regions responsible for learning and emotional control, leading to cognitive decline [84]. The seminal role of β-amyloid (Aβ) protein has been assessed in many AD studies [85,86]. Amyloid plaques resulting from the accumulation of Aβ proteins in the nerve cells set off a cascade of events that impair neurotransmission, leading to synaptic loss, which is the major cause of cognitive decline in AD patients [87]. Increased expression of NAD^+^ is associated with a decrease in the toxicity of Aβ oligomers in AD [88]. Treatment with the NAD^+^ precursor NMN improves cognitive function in Aβ oligomer AD model rats and attenuates neuronal cell death in organotypic hippocampal slices treated in Aβ oligomer culture media [88]. Supplementation with NMN in AD transgenic mice also attenuates mitochondrial respiratory deficits, Aβ production, and synaptic loss, and partially inhibits the activation of the JNK pathway and therefore might be a therapeutic option for AD [89,90]. Furthermore, preclinical studies show that treatment with NR reduces the formation of amyloid plaques, improves contextual memory and cognitive function, and attenuates synaptic plasticity by promoting PGC-1α-mediated β-secretase degradation, which prevents the production of Aβ in the brain of transgenic AD mouse models [68,91]. Supplementation with NR improves cognitive function, reverses DNA damage, and restores synaptic plasticity in the hippocampus of an AD mouse model (3 × TgAD/Polβ^+/−^) with introduction of a DNA repair deficiency [84]. Several studies have reported high cholesterol as a risk factor for developing AD later in life, as elevated cholesterol levels can promote the production of Aβ proteins [92]. Increased membrane cholesterol promotes Aβ-induced calpain activation, toxic 17 kDa tau production, and cell death in mature neurons [93]. In contrast, decreased membrane cholesterol reduces mature neurons' susceptibility to these Aβ-mediated cellular processes [93]. The deficiency of niacin in aging populations is also associated with dementia, a characteristic of AD. Niacin may offer protection in AD by decreasing serum and intracellular cholesterol levels [94]. Niacin also serves as a pharmacological agonist of GPR109A and has been reported to reduce the progression of atherosclerosis through GPR109A activation on immune cells, thus showing the potential of GPR109A in reducing inflammation [95]. Moreover, niacin is also produced by gut microbiota and its deficiency can lead to intestinal inflammation and pellagra [96]. A study showed that niacin supplementation was found to suppress colitis and colon cancer through the activation of GPR109A [97]. Furthermore, it is suggested that the induction of liver X receptors (LXR) might be associated with cholesterol-induced amyloid deposition in AD [98] as LXRs are key regulators of cholesterol and fatty acid metabolism. Specifically, LXR agonists can facilitate Aβ-42 clearance and inhibit the amyloid precursor protein's processing, thereby being a therapeutic option for AD [99]. Treatment with niacin increases the mRNA expression of LXRα and PPARγ and promotes cholesterol efflux in hypercholesterolemic rabbit adipocytes [100]. Several studies have reported a decline in levels of *NMNAT2* before neurodegeneration in mouse models of dementia, Parkinsonism-17, and AD [101,102]. In addition to its role in NAD^+^ synthesis, NMNAT2 acts as a chaperone and aids in the clearance or refolding of misfolded Tau aggregates, a characteristic of AD, by forming a complex with heat shock protein 90 (HSP90), thereby reducing proteotoxic stress to maintain neuronal health [103]. Deletion of NMNAT2 is found to increase the vulnerability of cortical neurons to proteotoxic stress and excitotoxicity [103]. Oral NAM has been found to selectively reduce phosphoThr231-tau and restore cognition in an AD mouse model through a mechanism similar to that of SIRT1 inhibition [102]. An ex vivo study showed that NAM treatment reduced lipid peroxidation, ROS production, and protein oxidation and improved mitochondrial reduction capacity against Aβ (1–42) in rat synaptosomes [104]. In contrast, a study demonstrated that niacin-deficient rats showed decreased NAD^+^ and intracellular cyclic ADP-ribose (cADPR) levels and improved spatial learning ability [105]. However, niacin-supplemented rats showed increased NAD^+^ and cADPR levels and an impaired spatial learning ability in a Morris water maze test [105]. The deletion of CD38, an NAD^+^ glycohydrolase, in an AD mouse model reduced Aβ plaque load and improved spatial learning, suggesting a role of CD38 inhibition in treating AD [106].

### 5.2 Parkinson's disease

PD is a progressive neurodegenerative disorder characterized by both motor (resting tremors, bradykinesia, and muscular rigidity) and non-motor features due to the loss of striatal dopaminergic and non-dopaminergic neurons [107,108]. The core pathological process in PD is the loss of dopaminergic neurons in the substantia nigra, which leads to the depletion of dopamine in the striatum region, and these changes are associated with bradykinesia [109,110]. The NAD^+^/NADH ratio and NAD/NADP ratio, also known as the niacin index, are significantly reduced in PD patients [111]. Supplementation of low-dose niacin was found to modulate the niacin index, GPR109A, and improve motor and cognitive functions in a PD patient with no side effects [112]. Niacin supplementation was also found to improve PD symptoms such as rigidity and bradykinesia, but a high dose of niacin resulted in side effects such as nightmares and skin rashes in a PD patient [113]. A case study also reported supplementation with NAD^+^ improved cellular resilience to dopaminergic neuronal loss in a patient with PD [114]. NAM plays both neuroprotective and neurotoxic roles in PD [115]. Supplementation with NAM rescues mitochondrial defects in *Parkin* and *PINK1* models of PD [116,117]. A recent study by Schӧndorf et al. reported that supplementation with NR restored mitochondrial function in neurons obtained from stem cells of a patient with PD and that NR prevented motor decline and loss of dopaminergic neurons in fly models of PD [118]. SIRT1 was downregulated in postmortem tissue samples from patients with PD, and overexpression of SIRT1 protected SH-SY5Y cells from toxin-induced cell death and reduced the formation of α-synuclein aggregates [119].

### 5.3 Huntington's disease

Huntington's disease (HD) is a progressive autosomal-dominant neurodegenerative disorder caused by the expansion of glutamine repeats in huntingtin protein (HTT) [120]. HD is characterized by cognitive decline, mood alterations, and repetitive involuntary choreiform movements caused by the degeneration of striatal spiny neurons [77]. Oxidative stress and mitochondrial dysfunction are the main cellular features of patients with HD. Neuroactive metabolites in the KYN pathway generated by the degradation of tryptophan are implicated in the pathogenesis of neurodegenerative diseases, including HD [121,122]. It is suggested that NR can enhance mitochondrial function via the SIRT1 and SIRT3 PGC-1α pathway and could also delay the translocation of mutant Htt into the nucleus, demonstrating that it is a potential pharmacologic agent for treating HD [123]. A study also showed that reduction in SIRT2 levels leads to increased survival of photoreceptor neurons, thereby exhibiting a neuroprotective effect in Httex1p Q93-expressing fly models of HD [124]. However, brain-specific knockout of SIRT1 worsens brain pathology, whereas SIRT1 overexpression improves survival and expression of the brain-derived neurotrophic factor in an HD mouse model [120]. As many studies have reported the neuroprotective effect of PARP inhibition in HD mouse models [80,125], the synergistic effect of PARPi with NR treatment needs to be explored in HD.

## 6 Role of NAD^+^ in metabolic disorders

In the last few decades, the increasing prevalence of metabolic disorders has added to the urban population's global health burden. Metabolic disorders include a cluster of risk factors such as obesity, insulin resistance, hypertension, and dyslipidemia, leading to the development of type 2 diabetes (T2D) and cardiovascular pathologies [126]. Levels of endogenous metabolites reflect the nutrient status in cells, and these levels determine the redox state and in turn are influenced by the redox state of NAD^+^ [127]. Levels of NAD^+^ directly influence metabolic enzymes' activity in various energy-production pathways and indirectly influence many more downstream of those effects. Regulation of the intracellular NAD^+^ pool seems to have a therapeutic potential in treating metabolic syndrome and modulates processes associated with the pathogenesis of obesity, non-alcoholic fatty liver disease (NAFLD), and T2D [128].

### 6.1 Diabetes

Increased NADH levels cause reductive stress, elevating cellular ROS levels and leading to insulin resistance, insulin deficiency, and cell death [129]. Thus, restoring NAD^+^ can help decrease the redox imbalance in diabetes. Altered or defective SIRT1 signaling mediated by NAD^+^ is implicated in insulin resistance and T2D, as SIRT1 positively regulates insulin signaling on multiple levels [130]. In addition, NAMPT has a crucial role in regulating insulin secretion in pancreatic β cells as it functions as an intra- and extracellular NAD biosynthetic enzyme [131]. A study showed that impaired glucose tolerance and reduced insulin secretion in pancreatic β cells of NAMPT^(+/−)^ heterozygous mice could be rescued by NMN administration [131]. This study implicated that maintaining NAD^+^ levels was important for pancreatic functionality. NMN also efficiently restored NAD^+^ levels by ameliorating glucose intolerance in high-fat diet (HFD)-induced T2D mice and further improved lipid profiles in age-induced T2D mice [43]. Another study demonstrated the neuroprotective effect of NR by administering NR to prediabetic and T2D mice. NR significantly improved glucose tolerance, reduced weight gain, reduced hepatic steatosis, and protected against sensory neuropathy in prediabetic mice and diabetic neuropathy in T2D mice [132]. Although replenishing NAD^+^ using NAM-related compounds appears to be successful in preclinical models, it should be noted that these compounds can also lead to an increase in NAM catabolites, which may act as uremic toxins, especially in patients with diabetes due to impaired NAM salvage pathway reactions [133]. Polyphenols, benfotiamine, aldose reductase inhibitors, acetyl-l-carnitine, or insulin sensitizers (in T2D) are all worthy of consideration as combinatorial therapies with NAD precursors to mitigate the effects of NAM catabolites [133]. A study found that nicotinamide treatment increased blood glucose and plasma N′-methylnicotinamide concentrations in rats. Exposure to N′-methylnicotinamide reduced NAD contents in the rat liver and muscle and increased levels of H_2_O_2_ in the rat plasma, suggesting a potential role of N′-methylnicotinamide in T2D [134].

**Figure 5:**
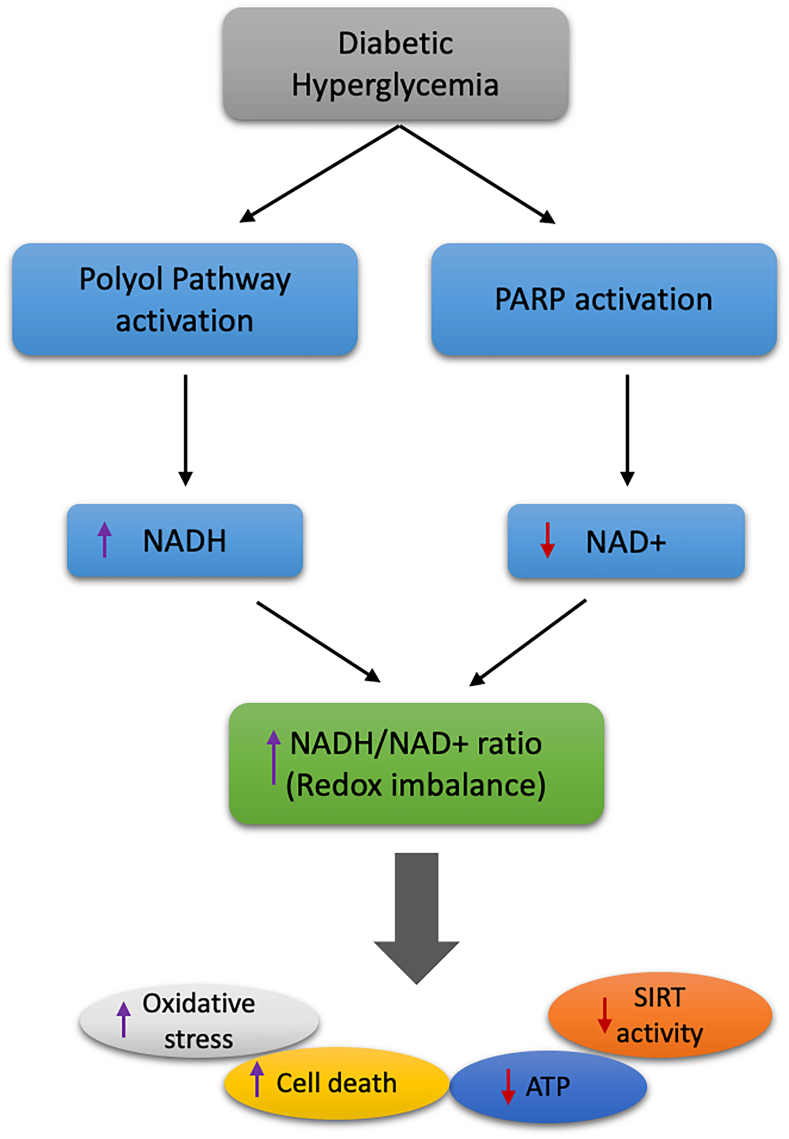
The polyol pathway becomes highly active during hyperglycemia, leading to the massive production of NADH [202,203]. Consumption of NAD^+^ by PARPs results in NAD^+^ decline. During diabetes, accumulation of NADH and depletion of NAD^+^ leads to an increase in redox imbalance, which causes oxidative stress, decreased SIRT activity, decreased ATP production, and increased cell death.

Moreover, the clearance of N′-methylnicotinamide is found to be slow in patients with diabetes [134]. Under hyperglycemic conditions, glucose flux into the glycolytic pathway, Krebs cycle, and polyol pathway may increase, resulting in the overproduction of NADH, which leads to an increase in ROS due to an overload in mitochondrial NADH, thereby contributing to insulin resistance [129] (Figure 5). Inhibition of mitochondrial electron transport chain complex I by rotenone, amobarbital, and NDUFA13 knockdown helped improve glucose homeostasis independently of AMPK activation in diabetes [135]. Moreover, inhibition of complex I by rotenone resulted in increased NADH levels and reduced cellular NAD^+^/NADH ratios, thus showing the efficacy of inhibiting complex I for the alleviation of hyperglycemia [135]. In such cases, several anti-hyperglycemic drugs such as metformin, thiazolidinediones, berberine, and rotenone have shown glucose-lowering effects with improved glucose tolerance and insulin sensitivity [135]. A clinical study showed that there was no effect of NR supplementation on insulin sensitivity and endogenous glucose production in obese insulin-resistant men [136].

### 6.2 Obesity

Obesity reduces enzymatic activities in the mitochondria, promotes metabolic inflexibility, and is associated with T2D and cardiovascular diseases. Obesity is associated with altered mitochondrial and NAD^+^ homeostasis in adipose tissues, resulting in the enlargement of adipose tissues, making them the most plastic organ in the body [137]. Adipocytes help regulate and maintain energy metabolism, thereby preserving mitochondrial function. An excess in energy substrates can lead to mitochondrial dysfunction, with alterations in lipid and glucose metabolism, contributing to the development of metabolic disorders [138]. Biosynthesis of NAD^+^ in the adipose tissue depends on the expression of NAMPT, and the expression of NAMPT is reduced in the adipose tissue of obese patients [139]. Although the function and physiological relevance of the extracellular form of NAMPT (eNAMPT) remain controversial, a recent preclinical study observed that secretion of eNAMPT is mediated by SIRT1-dependent deacetylation in adipocytes, which promotes the regulation of hypothalamic NAD^+^ levels by supplying NMN to the hypothalamus [140]. Another preclinical study found that extracellular vesicle-contained eNAMPT promotes NAD^+^ biosynthesis, counteracts aging, and extends life span in aged mice [141]. However, the absence of adipose NAMPT can cause adipose tissue fibrosis, alter adipose tissue plasticity, and reduce mitochondrial respiratory capacity [142].

### 6.3 Hepatic steatosis and non-alcoholic fatty liver disease

Although NAMPT enhances the levels of NAD^+^ in hepatocytes, they can maintain a substantial level of NAD^+^ in the absence of NAMPT through the de novo synthesis pathway [143]. Primary hepatocytes can maintain the mitochondrial NAD^+^ pool independent of NAMPT, suggesting that mitochondrial NAD^+^ is less affected by the absence of NAMPT than by cytosolic or nuclear NAD^+^ [143]. Supplementation with NAD^+^ precursors such as NR improves mitochondrial functions in the liver and prevents hepatic lipid accumulation in high-fat diet-induced obesity [64]. The overexpression of NNMT induces hepatic steatosis and fibrosis and decreases liver NAD^+^ in mice fed a high-fat diet containing NAM, thus contributing to fatty liver disease [27].

Members of the sirtuin family are associated with inflammation and energy metabolism and play an important role in maintaining metabolic homeostasis [144]. Activation of SIRT1 protects against metabolic damage induced by a high-fat diet, along with improved glucose tolerance and protection against hepatic steatosis [145]. Treatment with resveratrol, a natural phenol, improves mitochondrial function and aerobic capacity in mice by activating SIRT1 and PGC-1α, a regulator of cellular energy metabolism [146]. Many preclinical and clinical studies showed that most SIRT genes are expressed in the white adipose tissue (WAT) [137]. The expression of SIRT1, SIRT2, SIRT3, SIRT4, and SIRT6 is downregulated in the WAT of HFD-fed rodents [[147], [148], [149]]. However, in contrast, preclinical studies showed that calorie restriction or nutrient depletion upregulates SIRT1 and SIRT2, whereas SIRT4 expression is downregulated in the WAT [[150], [151], [152], [153]]. Furthermore, hepatic steatosis primarily develops due to NAFLD progression, which leads to increased ROS and mitochondrial dysfunction due to ectopic lipid accumulation [154]. Reduced levels of NAD^+^ in the liver and NAMPT in the visceral adipose tissue are associated with the degree of steatosis in NAFLD [139,155]. In addition, there is direct evidence of the downregulation of SIRT1, SIRT3, SIRT5, and SIRT6 in patients with NAFLD [156]. Moreover, telomere shortening or dysfunction can repress sirtuin function and lead to liver fibrosis; however, intriguingly, supplementation of NMN, a precursor of NAD^+^, helps improve mitochondrial function and rescue liver fibrosis [157]. Several studies showed that overexpression of SIRT1 and NR helps restore diet-induced hepatic steatosis and mitochondrial dysfunction by elevating NAD^+^ levels [64,158,159]. However, another study demonstrated that NAM supplementation protects hepatocytes against palmitate-induced cell death by activating autophagy [160]. This suggests that SIRT1, NAM, and NAD^+^ precursors may be potential therapeutic options for treating NAFLD.

### 6.4 Kidney diseases

Several studies indicated that reduced levels of NAD^+^ and sirtuins are associated with a plethora of renal diseases, and augmentation or replenishment of NAD^+^ can help treat chronic kidney diseases [161]. NAD^+^ supplementation has been found to attenuate mesangial hypertrophy induced by high glucose by activating SIRT1 and SIRT3, which block pro-hypertrophic AKT signaling and enhance the activity of anti-hypertrophic AMPK signaling in mesangial cells, thus preventing the induction of mTOR-mediated protein synthesis [162]. As aged kidneys have decayed NAD^+^ metabolism and reduced levels of SIRT1, supplementation with NMN can efficiently restore NAD^+^ levels and protect aged mice against cisplatin-induced acute kidney injury (AKI) [163]. Moreover, the occurrence of AKI mostly depends on the mitochondrial biogenesis regulator PGC-1α, which is a critical determinant of renal recovery from ischemic injury [164]. Supplementation with NAM can effectively increase NAD^+^ levels and renal function in postischemic mice [164]. A preclinical study showed that cotreatment with cisplatin and β-lapachone significantly increased intracellular NAD^+^ levels and attenuated cisplatin-induced AKI in mice due to the activation of NAD(P)H:quinone oxidoreductase 1 (NQO1) by β-lapachone [165]. A clinical study demonstrated that oral administration of NAM increased circulating NAD^+^ metabolites associated with less AKI [166].

## 7 NAD^+^ in cancer

**Figure 6:**
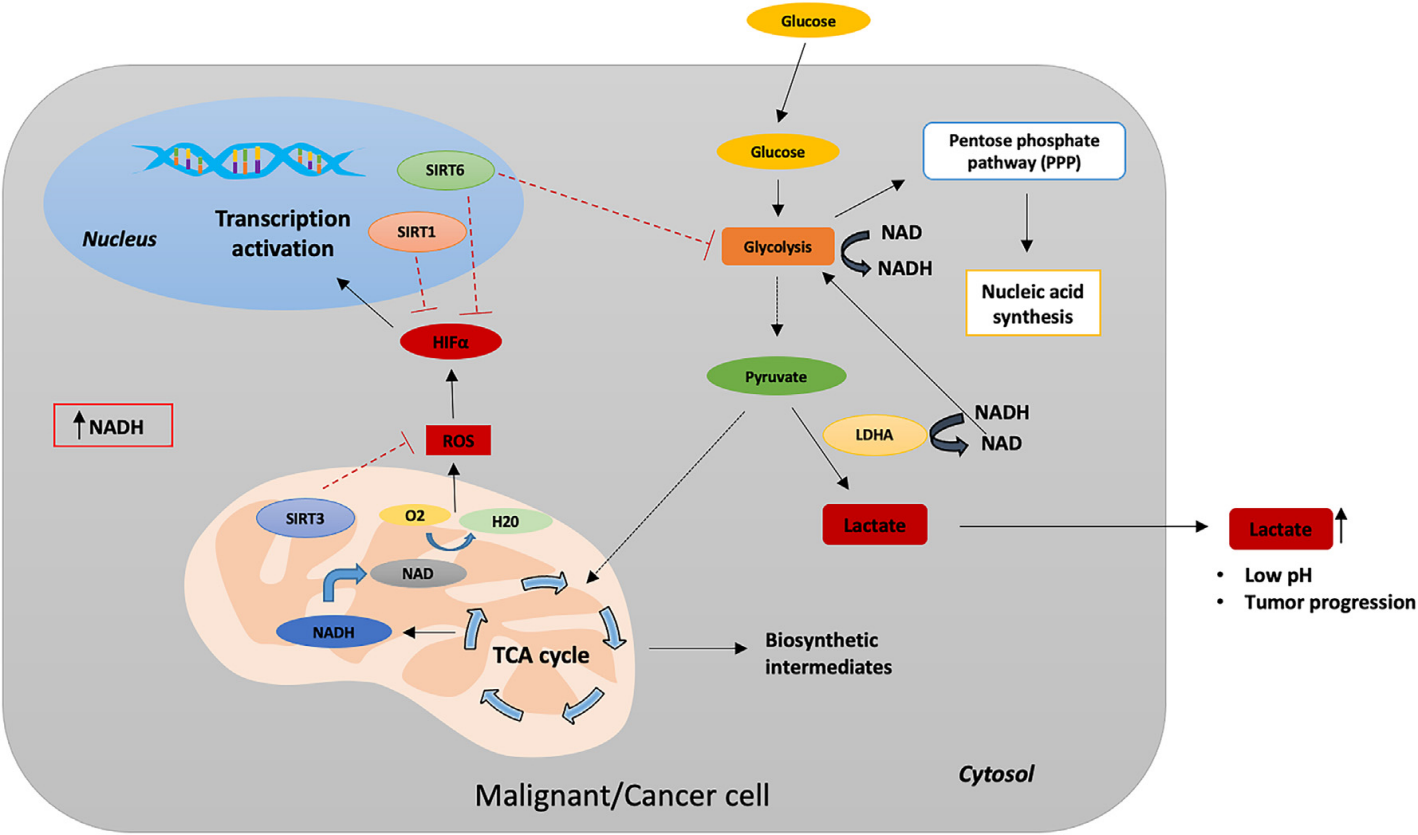
NAD metabolism in cancer cells. Cancer cells rely on increased glycolysis rates for energy production and regenerate NAD^+^ by converting accumulating pyruvate into lactate to maintain glycolysis. Excess lactate accumulation in tumor cells increases the level of NADH relative to NAD and perturbs the NAD/NADH balance in cells [204]. In contrast, SIRT1 acts as an inactivator of HIFα and prevents its nuclear translocation. SIRT6 acts as a corepressor of HIFα to prevent the transcriptional process, and mitochondrial-localized SIRT3 suppresses ROS production.

Cancer cells prefer aerobic glycolysis over OXPHOS, even in the presence of sufficient oxygen for energy metabolism [167] due to increased glucose uptake and glycolytic flux but reduced activity of the pyruvate dehydrogenase enzyme, which results in reduced pyruvate to acetyl CoA [168]. However, recent studies demonstrated that OXPHOS is upregulated in certain cancers [169]. Enhanced glycolysis is characterized by increased nutrient uptake and increased protein, lipid, and nucleic acid synthesis rates to support high rates of cancer cell proliferation (Figure 6). Elevated levels of NAD^+^ augment the process of anaerobic glycolysis through glyceraldehyde 3-phosphate dehydrogenase (GAPDH) and lactate dehydrogenase (LDH) and contribute to cancer cell proliferation [170].

**Figure 7:**
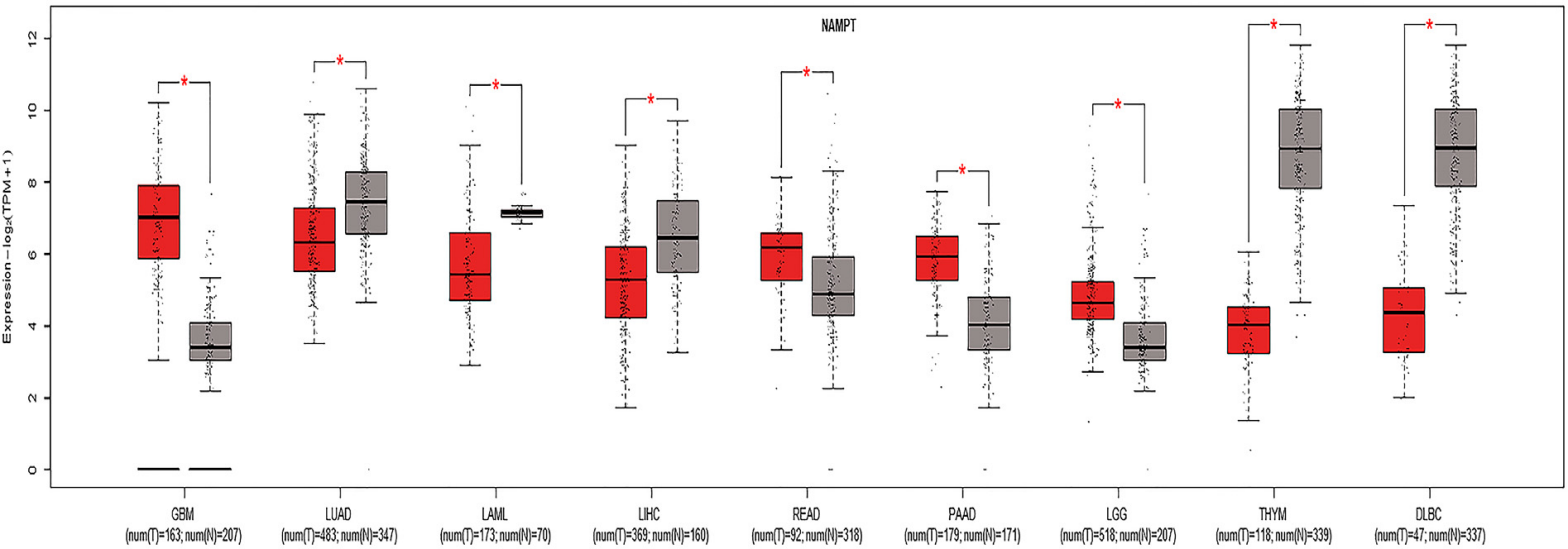
Expression profiles of NAMPT in nine cancer types and corresponding normal tissues per the TCGA-GTEx GEPIA2 dataset.

**Figure 8:**
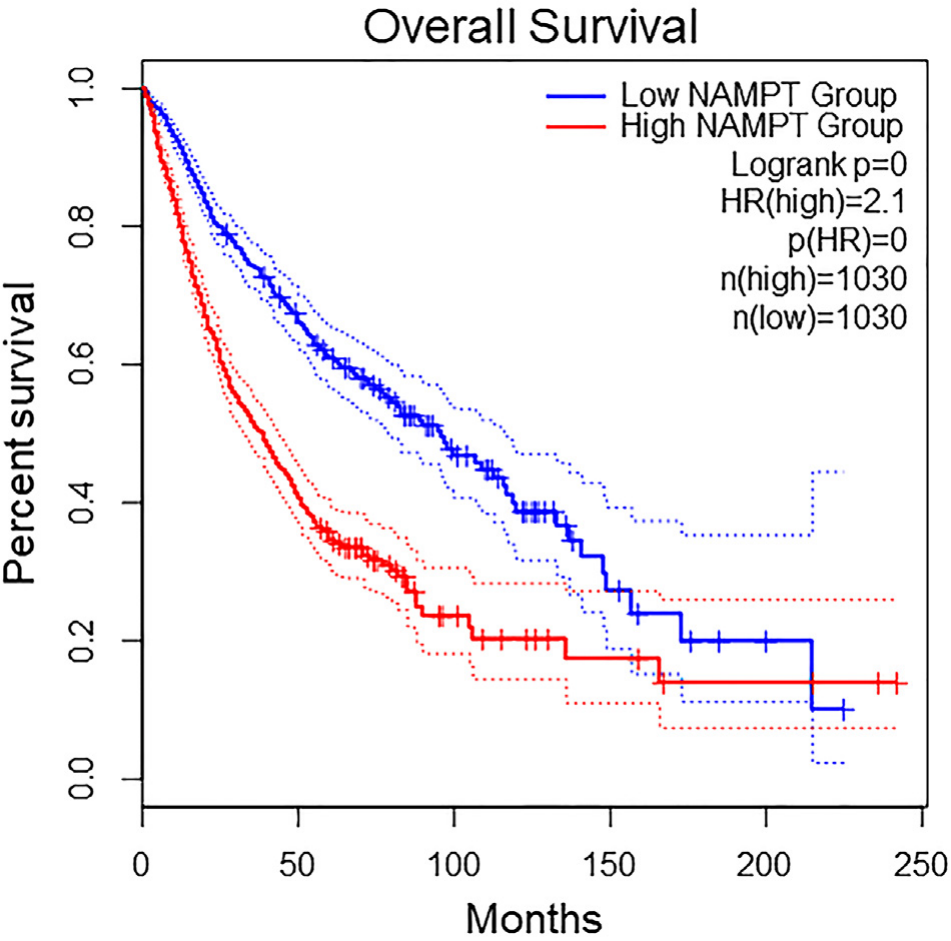
Overall survival rates of patients in low- and high-NAMPT expression groups in nine cancer types (GBM, LUAD, LAML, LIHC, READ, PAAD, LGG, THYM, and DLBC) using the TCGA-GTEx GEPIA2 dataset.

NAMPT is overexpressed in many types of cancers [171]. Overexpression of NAMPT accompanied by increased levels of NAD^+^ promotes cancer cell survival by making cells resistant to anti-cancer reagents. Expression profiles of NAMPT in nine different cancer types are shown in Figure 7. The well-known oncogene *c-MYC* regulates the expression of NAMPT and enhances the process of glycolysis and lactate production, leading to Warburg effects [172,173]. Furthermore, NAMPT-specific inhibitors reduce NAD^+^ levels by inhibiting energy metabolism pathways such as glycolysis, citric acid cycle, and OXPHOS, contributing to the suppression of cancer cell proliferation. Studies also reported the association of eNAMPT with the progression of cancers such as leukemia, hepatocellular carcinoma, and melanoma [[174], [175], [176], [177]] and in the promotion of cancer-related inflammation and epithelial to mesenchymal transition [178]. NAD^+^ also regulates DNA repair mechanisms, stress responses, and gene expression by serving as a substrate for enzymes such as sirtuin, PARP, and NAD glycohydrolase [170]. An interesting study showed that restoring NAD^+^ pools with NR can prevent DNA damage and tumor formation induced by unconventional prefoldin RPB5 interactor (URI) in hepatocellular carcinoma [179]. Overall survival rates in high- and low-NAMPT expression groups in nine cancer types are shown in Figure 8. NAPRT, a key enzyme mediating NAD biosynthesis from nicotinic acid, plays an important role in cancer cell metabolism. Studies reported downregulation of the *NAPRT* gene in cancers such as glioblastoma and neuroblastoma [180,181]. A recent study reported overexpression of the *NAPRT* gene in a subset of common types of cancer such as breast, prostate, liver, pancreatic, ovarian, and head and neck cancers, where it promotes cancer cell metabolism and reduces the susceptibility to NAMPT inhibitors and DNA-damaging drugs [182]. A recent study showed that NAPRT silencing reduces the NAD^+^ pool in human ovarian and pancreatic cancer cells and sensitizes cells to NAMPT inhibitors [182]. Silencing of NAPRT in cancer cells reduces OXPHOS, protein synthesis, and ATP levels due to a reduction in mitochondrial NADH [182]. Thus, NAPRT could prove beneficial to understanding the function of NAD^+^ biosynthetic mechanisms in cancer and likely serve as a potential therapeutic target. PARPs are major mediators of cellular responses associated with DNA repair and genomic stability. Activation of PARP in cells with DNA damage or external stressors leads to NAD depletion, as PARP consumes large amounts of NAD^+^ during DNA repair processes [183]. PARPi is an effective therapeutic agent against BRCA1- and BRCA2-associated cancers, as BRCA mutations lead to DNA double-strand breaks that cannot be efficiently repaired, thus leading to the death of cancer cells. The regulation of PARP1 activity has a significant impact on the metabolism of NAD^+^ [184]. The endogenous PARP1 inhibitor macroH2A1.1 decreases the consumption of NAD^+^ in differentiating cells and increases the availability of mitochondrial NAD^+^, thereby enabling increased OXPHOS [184]. However, PARPi is only effective against certain cancers. The combination of PARPi and β-lapachone, an NAD(P)H:quinone oxidoreductase 1 (NQO1) bioactivatable drug, has synergistic anti-tumor activity and blocks PARP-dependent DNA repair in NQO1-overexpressing cancers such as non-small cell lung cancer, breast cancer, and pancreatic cancer, causing tumor-selective apoptosis [185]. However, any disturbances in levels of NAD^+^ influence the suppression of tumor formation mediated by sirtuins. NAD^+^ and NAMPT are involved in the activation of SIRT1 [186]. NAMPT controls the activity of SIRT1 by regulating NAD^+^ levels. The overexpression of NAMPT increases survival in a SIRT1-dependent manner, whereas inhibition of NAMPT contributes to premature senescence [187]. SIRT1 with NAMPT is overexpressed in many cancers such as colorectal cancer, prostate cancer, and gliomas [[188], [189], [190]]. Moreover, mitochondrial NAD^+^ is a cosubstrate for mitochondrial-localized SIRT3, SIRT4, and SIRT5. The overexpression of nicotinamide nucleotide transhydrogenase (NNT), a mitochondrial enzyme involved in the generation of NADPH from NADH and found to enhance anti-oxidant capacity, induces differentiation and reduces clonogenicity of glioblastoma tumor-initiating cells [191], whereas a reduction in NAD^+^ facilitates metastasis of hepatocellular carcinoma cells [192]. SIRT2 is overexpressed in several cancers such as gastric cancer [193] and hepatocellular carcinoma [194]. SIRT2 enhances NADPH production and promotes leukemia cell proliferation by deacetylation and activation of glucose-6-phosphate dehydrogenase [195]. Consumption of large amounts of intracellular NAD^+^ by PARPs reduces the availability of NAD^+^ for sirtuins, which can lead to aberrant deacetylation of tumor suppressor proteins such as p53 [196]. As p53 is essential for the effective regulation of different cellular processes such as apoptosis, autophagy, and senescence, p53 mutations can result in cancer cell growth by enabling cancer cell survival under nutrient-limiting conditions [197]. Additionally, the expression of p53 depends on the concentration of NAD^+^.

## 8 Future directions

Modern sedentary lifestyles are major factors contributing to the development of age-related neurodegenerative disorders. Exercise and dietary measures (calorie restriction) may prevent or delay the development of age-related neurological disorders. Emerging evidence from many preclinical and clinical studies has deepened our understanding of the role of NAD^+^- and NAD^+^-dependent enzymes in aging, synaptic plasticity, neurodegenerative disorders, and cancer. NAD^+^ depletion is accentuated in aging, and many neurodegenerative disorders such as AD, PD, and the augmentation of NAD^+^ have proven beneficial in mitigating the pathological features in preclinical models of these disorders. Although the supplementation of NAD^+^ precursors is beneficial for augmenting the levels of NAD^+^, it can be limited due to their variable cell permeability, stability, dosage, and side effects. For instance, a study demonstrated the possible adverse effects of high doses of NAM, a precursor of NAD+, and suggested that high doses of NAM can cause genomic instability, reduce cellular methyl pools, and cause insulin resistance through methylated NAM [198]. Moreover, niacin, a NAD + precursor, was associated with mild or moderate flushing in many clinical trial studies [199]. However, this was due to its interaction with GPR109A rather than NAD^+^ per se, and the severity and frequency of the flushing was found to decrease with continued niacin treatment [199].

Many unanswered questions relating to NAD^+^ need to be further explored. First, the NAD^+^/NADH ratio of NAD^+^/NADH is complicated due to its varied distribution in different locations within the cell, and the direct role of impaired NAD^+^-dependent processes in humans remains unestablished. In this regard, longitudinal studies are needed to quantify NAD^+^ and its related metabolites, including the quantification of NAD^+^ and related metabolites in different disease conditions. Second, the application of NAMPT inhibitors as an anti-cancer agent in humans remains challenging. Although inhibiting NAMPT has been effective in cancer cells as it attenuates glycolysis and activates autophagy processes, complete inhibition or depletion of NAMPT can cause motor neuron degeneration, motor function deficits, mitochondrial dysfunction, and defective synaptic function in mice [200]. Therefore, it is necessary to select patients who efficiently adapt to NAMPT inhibitor therapy.

The synergistic effect of DNA-damaging reagents and NAMPT inhibitors might help induce apoptosis in cancer cells, as the DNA damage process can consume large amounts of NAD^+^. Modulation of NAD^+^ levels must be considered with caution, as high levels of NAD^+^ enhance glycolysis, which promotes cancer cell proliferation and survival, thereby making NAD^+^ a potential contributing factor to tumorigenesis. Therefore, an anti-cancer clinical strategy with individual patient profiling is necessary to identify high-efficacy treatments. Thus, more preclinical studies are required to understand the mechanisms of NAD^+^ replenishment in aging and neurodegenerative and cancer models to understand the contribution of NAD^+^ to these morbidities. To date, administering NAD^+^ precursors is well-tolerated in humans, but their safety remains undetermined. Therefore, more clinical trials are required to assess the safety and efficacy of NAD^+^ precursors in aging and various neurological and metabolic disorders.

## 9 Conclusions

It has been established that an alteration in the NAD^+^/NADH ratio can lead to derailment of the biological system and contribute to various neurodegenerative disorders, aging, and tumorigenesis. DNA damage can lead to PARP activation and cause reductions in NAD^+^ pools due to perturbations in NAD^+^ biosynthesis or consumption of NAD^+^ by NAD^+^-consuming enzymes. Increasing evidence shows the feasibility of using NAD^+^ precursors and intermediates to boost the levels of NAD^+^ to limit aging and neurodegenerative processes. However, the therapeutic potential of using NAD^+^ precursors for cancer treatment remains inconclusive. Therefore, to better characterize the dynamics of NAD^+^ homeostasis in different diseases, future research should focus on measuring the fluxes through pathways associated with NAD^+^ synthesis and degradation, as every tissue in the body has its NAD^+^ metabolome.

## Abbreviations

NR: nicotinamide riboside
NA: nicotinic acid
NAM: nicotinamide
Trp: tryptophan
NAMN: nicotinic acid mononucleotide
NAPRT: nicotinic acid phosphoribosyltransferase
NAAD: nicotinic acid adenine dinucleotide
NMN: nicotinamide mononucleotide
NMNAT: nicotinamide mononucleotide adenylyl transferase
NADSYN: NAD synthase
NRK: nicotinamide riboside kinase
QA: quinolinic acid
NAMPT: nicotinamide phosphoribosyltransferase
PARP: poly (ADP-ribose) polymerase
OXPHOS: oxidative phosphorylation
ETC: electron transport chain
Trp: tryptophan
NMN: nicotinamide mononucleotide
PRPP: phosphoribosyl pyrophosphate
NADH: nicotinamide adenine dinucleotide reduced form
PGC-1α: proliferator-activated receptor-γ coactivator 1
ATP: adenosine triphosphate
NADPH: nicotinamide adenine dinucleotide phosphate hydrogen
ROS: reactive oxygen species
GBM: glioblastoma multiforme
LUAD: lung adenocarcinoma
LAML: acute myeloid leukemia
LIHC: liver hepatocellular carcinoma
READ: rectum adenocarcinoma
PAAD: pancreatic adenocarcinoma
LGG: lower grade glioma
THYM: thymoma
DLBC: diffuse large B cell lymphoma
URI: unconventional prefoldin RPB5 interactor
KYN: kynurenine
QA: quinolinic acid
NFK: *N*-formylkynurenine
IDO: indoleamine-2,3-dioxygenase
TDO: tryptophan-2,3-dioxygenase
3-HAA: 3-hydroxyanthranilic acid
ACMS: α-amino-β-carboxymuconate-ε-semialdehyde
TFAM: transcriptional factor A, mitochondria
AD: Alzheimer's disease
ISCs: intestinal stem cells
AKI: acute kidney injury
LXR: liver X receptors
Aβ: β-amyloid
HD: Huntington's disease
HTT: huntingtin protein
MRS: magnetic resonance spectroscopy
T2D: type 2 diabetes
NAFLD: non-alcoholic fatty liver disease

## References

[1] Kane Alice E., Sinclair David A. (2018). Sirtuins and NAD+ in the development and treatment of metabolic and cardiovascular diseases. Circulation Research.

[2] Xiao W., Wang R.-S., Handy D.E., Loscalzo J. (2018). NAD(H) and NADP(H) redox couples and cellular energy metabolism. Antioxidants and Redox Signaling.

[3] Heikal A.A. (2010). Intracellular coenzymes as natural biomarkers for metabolic activities and mitochondrial anomalies. Biomarkers in Medicine.

[4] Trisolini L., Gambacorta N., Gorgoglione R., Montaruli M., Laera L., Colella F. (2019). FAD/NADH dependent oxidoreductases: from different amino acid sequences to similar protein shapes for playing an ancient function. Journal of Clinical Medicine.

[5] Xie N., Zhang L., Gao W., Huang C., Huber P.E., Zhou X. (2020). NAD+ metabolism: pathophysiologic mechanisms and therapeutic potential. Signal Transduction and Targeted Therapy.

[6] Wallace D.C. (2012). Mitochondria and cancer. Nature Reviews Cancer.

[7] Ying W. (2008). NAD+/NADH and NADP+/NADPH in cellular functions and cell death: regulation and biological consequences. Antioxidants and Redox Signaling.

[8] Pehar M., Harlan B.A., Killoy K.M., Vargas M.R. (2018). Nicotinamide adenine dinucleotide metabolism and neurodegeneration. Antioxidants and Redox Signaling.

[9] Demarest T.G., Babbar M., Okur M.N., Dan X., Croteau D.L., Fakouri N.B. (2019). NAD+ metabolism in aging and cancer. Annual Review of Cancer Biology.

[10] Nikiforov A., Kulikova V., Ziegler M. (2015). The human NAD metabolome: functions, metabolism and compartmentalization. Critical Reviews in Biochemistry and Molecular Biology.

[11] Rajman L., Chwalek K., Sinclair D.A. (2018). Therapeutic potential of NAD-boosting molecules: the in vivo evidence. Cell Metabolism.

[12] Aman Y., Qiu Y., Tao J., Fang E.F. (2018). Therapeutic potential of boosting NAD+ in aging and age-related diseases. Translational Medicine of Aging.

[13] Lau C., Niere M., Ziegler M. (2009). The NMN/NaMN adenylyltransferase (NMNAT) protein family. Frontiers in Bioscience.

[14] Emanuelli M., Carnevali F., Saccucci F., Pierella F., Amici A., Raffaelli N. (2001). Molecular cloning, chromosomal localization, tissue mRNA levels, bacterial expression, and enzymatic properties of human NMN adenylyltransferase. Journal of Biological Chemistry.

[15] Yalowitz J.A., Xiao S., Biju M.P., Antony A.C., Cummings O.W., Deeg M.A. (2004). Characterization of human brain nicotinamide 5'-mononucleotide adenylyltransferase-2 and expression in human pancreas. Biochemical Journal.

[16] Berger F., Lau C., Dahlmann M., Ziegler M. (2005). Subcellular compartmentation and differential catalytic properties of the three human nicotinamide mononucleotide adenylyltransferase isoforms. Journal of Biological Chemistry.

[17] Raffaelli N., Sorci L., Amici A., Emanuelli M., Mazzola F., Magni G. (2002). Identification of a novel human nicotinamide mononucleotide adenylyltransferase. Biochemical and Biophysical Research Communications.

[18] Hikosaka K., Ikutani M., Shito M., Kazuma K., Gulshan M., Nagai Y. (2014). Deficiency of nicotinamide mononucleotide adenylyltransferase 3 (nmnat3) causes hemolytic anemia by altering the glycolytic flow in mature erythrocytes. Journal of Biological Chemistry.

[19] Yang H., Yang T., Baur J.A., Perez E., Matsui T., Carmona J.J. (2007). Nutrient-sensitive mitochondrial NAD+ levels dictate cell survival. Cell.

[20] Nikiforov A., Dölle C., Niere M., Ziegler M. (2011). Pathways and subcellular compartmentation of NAD biosynthesis in human cells: from entry of extracellular precursors to mitochondrial NAD generation. Journal of Biological Chemistry.

[21] Marletta A.S., Massarotti A., Orsomando G., Magni G., Rizzi M., Garavaglia S. (2015). Crystal structure of human nicotinic acid phosphoribosyltransferase. FEBS Open Bio.

[22] Belenky P., Bogan K.L., Brenner C. (2007). NAD+ metabolism in health and disease. Trends in Biochemical Sciences.

[23] Magni G., Amici A., Emanuelli M., Orsomando G., Raffaelli N., Ruggieri S. (2004). Enzymology of NAD+ homeostasis in man. Cellular and Molecular Life Sciences.

[24] Houtkooper R.H., Cantó C., Wanders R.J., Auwerx J. (2010). The secret life of NAD+: an old metabolite controlling new metabolic signaling pathways. Endocrine Reviews.

[25] Kulkarni C.A., Brookes P.S. (2019). Cellular compartmentation and the redox/non-redox functions of NAD+. Antioxidants and Redox Signaling.

[26] Katsyuba E., Mottis A., Zietak M., De Franco F., van der Velpen V., Gariani K. (2018). De novo NAD(+) synthesis enhances mitochondrial function and improves health. Nature.

[27] Komatsu M., Kanda T., Urai H., Kurokochi A., Kitahama R., Shigaki S. (2018). NNMT activation can contribute to the development of fatty liver disease by modulating the NAD+metabolism. Scientific Reports.

[28] Kellenberger E., Kuhn I., Schuber F., Muller-Steffner H. (2011). Flavonoids as inhibitors of human CD38. Bioorganic & Medicinal Chemistry Letters.

[29] Tarragó M.G., Chini C.C.S., Kanamori K.S., Warner G.M., Caride A., de Oliveira G.C. (2018). A potent and specific CD38 inhibitor ameliorates age-related metabolic dysfunction by reversing tissue NAD(+) decline. Cell Metabolism.

[30] Escande C., Nin V., Price N.L., Capellini V., Gomes A.P., Barbosa M.T. (2013). Flavonoid apigenin is an inhibitor of the NAD+ ase CD38: implications for cellular NAD+ metabolism, protein acetylation, and treatment of metabolic syndrome. Diabetes.

[31] Boslett J., Hemann C., Zhao Y.J., Lee H.C., Zweier J.L. (2017). Luteolinidin protects the postischemic heart through CD38 inhibition with preservation of NAD(P)(H). Journal of Pharmacology and Experimental Therapeutics.

[32] Mouchiroud L., Houtkooper R.H., Auwerx J. (2013). NAD+ metabolism: a therapeutic target for age-related metabolic disease. Critical Reviews in Biochemistry and Molecular Biology.

[33] Pirinen E., Cantó C., Jo Y.S., Morato L., Zhang H., Menzies K.J. (2014). Pharmacological Inhibition of poly(ADP-ribose) polymerases improves fitness and mitochondrial function in skeletal muscle. Cell Metabolism.

[34] Slade D. (2020). PARP and PARG inhibitors in cancer treatment. Genes & Development.

[35] Baldwin P., Likhotvorik R., Baig N., Cropper J., Carlson R., Kurmasheva R. (2019). Nanoformulation of talazoparib increases maximum tolerated doses in combination with Temozolomide for treatment of Ewing sarcoma. Frontiers in Oncology.

[36] Zha S., Li Z., Cao Q., Wang F., Liu F. (2018). PARP1 inhibitor (PJ34) improves the function of aging-induced endothelial progenitor cells by preserving intracellular NAD+ levels and increasing SIRT1 activity. Stem Cell Research & Therapy.

[37] Almeida G.S., Bawn C.M., Galler M., Wilson I., Thomas H.D., Kyle S. (2017). PARP inhibitor rucaparib induces changes in NAD levels in cells and liver tissues as assessed by MRS. NMR in Biomedicine.

[38] Cohen M.S. (2020). Interplay between compartmentalized NAD(+) synthesis and consumption: a focus on the PARP family. Genes & Development.

[39] McReynolds M.R., Chellappa K., Baur J.A. (2020). Age-related NAD(+) decline. Experimental Gerontology.

[40] Mills K.F., Yoshida S., Stein L.R., Grozio A., Kubota S., Sasaki Y. (2016). Long-Term administration of nicotinamide mononucleotide mitigates age-associated physiological decline in mice. Cell Metabolism.

[41] Zhang H., Ryu D., Wu Y., Gariani K., Wang X., Luan P. (2016). NAD⁺ repletion improves mitochondrial and stem cell function and enhances life span in mice. Science.

[42] Parker D., Sloane R., Pieper C.F., Hall K.S., Kraus V.B., Kraus W.E. (2019). Age-related adverse inflammatory and metabolic changes begin early in adulthood. Journals of Gerontology Series A: Biological and Medical Sciences.

[43] Yoshino J., Mills K.F., Yoon M.J., Imai S.-I. (2011). Nicotinamide mononucleotide, a key NAD(+) intermediate, treats the pathophysiology of diet- and age-induced diabetes in mice. Cell Metabolism.

[44] Yoshino J., Baur J.A., Imai S.I. (2018). NAD(+) intermediates: the biology and therapeutic potential of NMN and NR. Cell Metabolism.

[45] Ramsey K.M., Mills K.F., Satoh A., Imai S. (2008). Age-associated loss of Sirt1-mediated enhancement of glucose-stimulated insulin secretion in beta cell-specific Sirt1-overexpressing (BESTO) mice. Aging Cell.

[46] Thompson S.L., Compton D.A. (2011). Chromosomes and cancer cells. Chromosome Research : An International Journal on the Molecular, Supramolecular and Evolutionary Aspects of Chromosome Biology.

[47] Ramis M.R., Esteban S., Miralles A., Tan D.X., Reiter R.J. (2015). Caloric restriction, resveratrol and melatonin: role of SIRT1 and implications for aging and related-diseases. Mechanism of Ageing and Development.

[48] Fritze C.E., Verschueren K., Strich R., Easton Esposito R. (1997). Direct evidence for SIR2 modulation of chromatin structure in yeast rDNA. The EMBO Journal.

[49] Bryan S., Baregzay B., Spicer D., Singal P.K., Khaper N. (2013). Redox-inflammatory synergy in the metabolic syndrome. Canadian Journal of Physiology and Pharmacology.

[50] Jin K. (2010). Modern biological theories of aging. Aging and Disease.

[51] Camacho-Pereira J., Tarragó M.G., Chini C.C.S., Nin V., Escande C., Warner G.M. (2016). CD38 dictates age-related NAD decline and mitochondrial dysfunction through an SIRT3-dependent mechanism. Cell Metabolism.

[52] Scheibye-Knudsen M., Mitchell S.J., Fang E.F., Iyama T., Ward T., Wang J. (2014). A high-fat diet and NAD(+) activate Sirt1 to rescue premature aging in cockayne syndrome. Cell Metabolism.

[53] Fang E.F., Hou Y., Lautrup S., Jensen M.B., Yang B., SenGupta T. (2019). NAD(+) augmentation restores mitophagy and limits accelerated aging in Werner syndrome. Nature Communications.

[54] Gomes A.P., Price N.L., Ling A.J., Moslehi J.J., Montgomery M.K., Rajman L. (2013). Declining NAD(+) induces a pseudohypoxic state disrupting nuclear-mitochondrial communication during aging. Cell.

[55] Chistiakov D.A., Sobenin I.A., Revin V.V., Orekhov A.N., Bobryshev Y.V. (2014). Mitochondrial aging and age-related dysfunction of mitochondria. BioMed Research International.

[56] Xu C., Wang L., Fozouni P., Evjen G., Chandra V., Jiang J. (2020). SIRT1 is downregulated by autophagy in senescence and ageing. Nature Cell Biology.

[57] Sasaki T., Maier B., Bartke A., Scrable H. (2006). Progressive loss of SIRT1 with cell cycle withdrawal. Aging Cell.

[58] Maynard S., Fang E.F., Scheibye-Knudsen M., Croteau D.L., Bohr V.A. (2015). DNA damage, DNA repair, aging, and neurodegeneration. Cold Spring Harbor Perspectives in Medicine.

[59] Imai S.-i., Guarente L. (2014). NAD+ and sirtuins in aging and disease. Trends in Cell Biology.

[60] Nakahata Y., Sahar S., Astarita G., Kaluzova M., Sassone-Corsi P. (2009). Circadian control of the NAD+ salvage pathway by CLOCK-SIRT1. Science.

[61] Braidy N., Guillemin G.J., Mansour H., Chan-Ling T., Poljak A., Grant R. (2011). Age related changes in NAD+ metabolism oxidative stress and Sirt1 activity in wistar rats. PloS One.

[62] Mouchiroud L., Houtkooper R.H., Moullan N., Katsyuba E., Ryu D., Cantó C. (2013). The NAD(+)/Sirtuin pathway modulates longevity through activation of mitochondrial UPR and FOXO signaling. Cell.

[63] Bai P., Cantó C., Oudart H., Brunyánszki A., Cen Y., Thomas C. (2011). PARP-1 inhibition increases mitochondrial metabolism through SIRT1 activation. Cell Metabolism.

[64] Cantó C., Houtkooper R.H., Pirinen E., Youn D.Y., Oosterveer M.H., Cen Y. (2012). The NAD(+) precursor nicotinamide riboside enhances oxidative metabolism and protects against high-fat diet-induced obesity. Cell Metabolism.

[65] Igarashi M., Miura M., Williams E., Jaksch F., Kadowaki T., Yamauchi T. (2019). NAD+ supplementation rejuvenates aged gut adult stem cells. Aging Cell.

[66] Fang E.F., Lautrup S., Hou Y., Demarest T.G., Croteau D.L., Mattson M.P. (2017). NAD(+) in aging: molecular mechanisms and translational implications. Trends in Molecular Medicine.

[67] Das A., Huang G.X., Bonkowski M.S., Longchamp A., Li C., Schultz M.B. (2018). Impairment of an endothelial NAD+-H2S signaling network is a reversible cause of vascular aging. Cell.

[68] Gong B., Pan Y., Vempati P., Zhao W., Knable L., Ho L. (2013). Nicotinamide riboside restores cognition through an upregulation of proliferator-activated receptor-γ coactivator 1α regulated β-secretase 1 degradation and mitochondrial gene expression in Alzheimer's mouse models. Neurobiology of Aging.

[69] Martens C.R., Denman B.A., Mazzo M.R., Armstrong M.L., Reisdorph N., McQueen M.B. (2018). Chronic nicotinamide riboside supplementation is well-tolerated and elevates NAD(+) in healthy middle-aged and older adults. Nature Communications.

[70] Fang E.F., Kassahun H., Croteau D.L., Scheibye-Knudsen M., Marosi K., Lu H. (2016). NAD(+) replenishment improves lifespan and healthspan in ataxia telangiectasia models via mitophagy and DNA repair. Cell Metabolism.

[71] Fang E.F., Scheibye-Knudsen M., Brace L.E., Kassahun H., SenGupta T., Nilsen H. (2014). Defective mitophagy in XPA via PARP-1 hyperactivation and NAD(+)/SIRT1 reduction. Cell.

[72] Williams P.A., Harder J.M., Foxworth N.E., Cochran K.E., Philip V.M., Porciatti V. (2017). Vitamin B(3) modulates mitochondrial vulnerability and prevents glaucoma in aged mice. Science (New York, N.Y.).

[73] Procaccini C., Santopaolo M., Faicchia D., Colamatteo A., Formisano L., de Candia P. (2016). Role of metabolism in neurodegenerative disorders. Metabolism.

[74] Hikosaka K., Yaku K., Okabe K., Nakagawa T. (2019). Implications of NAD metabolism in pathophysiology and therapeutics for neurodegenerative diseases. Nutritional Neuroscience.

[75] Zhang F., Wang S., Gan L., Vosler P.S., Gao Y., Zigmond M.J. (2011). Protective effects and mechanisms of sirtuins in the nervous system. Progress in Neurobiology.

[76] Wang J., He Z. (2009). NAD and axon degeneration: from the Wlds gene to neurochemistry. Cell Adhesion & Migration.

[77] Lautrup S., Sinclair D.A., Mattson M.P., Fang E.F. (2019). NAD+ in brain aging and neurodegenerative disorders. Cell Metabolism.

[78] Schwarcz R., Pellicciari R. (2002). Manipulation of brain kynurenines: glial targets, neuronal effects, and clinical opportunities. Journal of Pharmacology and Experimental Therapeutics.

[79] Czapski G.A., Cieślik M., Wencel P.L., Wójtowicz S., Strosznajder R.P., Strosznajder J.B. (2018). Inhibition of poly(ADP-ribose) polymerase-1 alters expression of mitochondria-related genes in PC12 cells: relevance to mitochondrial homeostasis in neurodegenerative disorders. Biochimica et Biophysica Acta (BBA)-Molecular Cell Research.

[80] Cardinale A., Paldino E., Giampà C., Bernardi G., Fusco F.R. (2015). PARP-1 inhibition is neuroprotective in the R6/2 mouse model of Huntington's disease. PloS One.

[81] Sasaki Y., Vohra B.P.S., Lund F.E., Milbrandt J. (2009). Nicotinamide mononucleotide adenylyl transferase-mediated axonal protection requires enzymatic activity but not increased levels of neuronal nicotinamide adenine dinucleotide. Journal of Neuroscience : The Official Journal of the Society for Neuroscience.

[82] Gerdts J., Brace E.J., Sasaki Y., DiAntonio A., Milbrandt J. (2015). SARM1 activation triggers axon degeneration locally via NAD⁺ destruction. Science (New York, N.Y.).

[83] Sasaki Y., Nakagawa T., Mao X., DiAntonio A., Milbrandt J. (2016). NMNAT1 inhibits axon degeneration via blockade of SARM1-mediated NAD(+) depletion. eLife.

[84] Hou Y., Lautrup S., Cordonnier S., Wang Y., Croteau D.L., Zavala E. (2018). NAD(+) supplementation normalizes key Alzheimer's features and DNA damage responses in a new AD mouse model with introduced DNA repair deficiency. Proceedings of the National Academy of Sciences of the United States of America.

[85] Sadigh-Eteghad S., Sabermarouf B., Majdi A., Talebi M., Farhoudi M., Mahmoudi J. (2015). Amyloid-Beta: a crucial factor in alzheimer's disease. Medical Principles and Practice.

[86] Murphy M.P., LeVine H. (2010). Alzheimer's disease and the amyloid-beta peptide. Journal of Alzheimer's Disease : JAD.

[87] Ding Y., Zhao J., Zhang X., Wang S., Viola K.L., Chow F.E. (2019). Amyloid beta oligomers target to extracellular and intracellular neuronal synaptic proteins in alzheimer's disease. Frontiers in Neurology.

[88] Wang X., Hu X., Yang Y., Takata T., Sakurai T. (2016). Nicotinamide mononucleotide protects against β-amyloid oligomer-induced cognitive impairment and neuronal death. Brain Research.

[89] Yao Z., Yang W., Gao Z., Jia P. (2017). Nicotinamide mononucleotide inhibits JNK activation to reverse Alzheimer disease. Neuroscience Letters.

[90] Long A.N., Owens K., Schlappal A.E., Kristian T., Fishman P.S., Schuh R.A. (2015). Effect of nicotinamide mononucleotide on brain mitochondrial respiratory deficits in an Alzheimer's disease-relevant murine model. BMC Neurology.

[91] Sorrentino V., Romani M., Mouchiroud L., Beck J.S., Zhang H., D'Amico D. (2017). Enhancing mitochondrial proteostasis reduces amyloid-β proteotoxicity. Nature.

[92] Sjögren M., Blennow K. (2005). The link between cholesterol and Alzheimer's disease. World Journal of Biological Psychiatry.

[93] Nicholson A.M., Ferreira A. (2009). Increased membrane cholesterol might render mature hippocampal neurons more susceptible to beta-amyloid-induced calpain activation and tau toxicity. Journal of Neuroscience.

[94] Chen J., Chopp M. (2010). Niacin, an old drug, has new effects on central nervous system disease. The Open Drug Discovery Journal.

[95] Lukasova M., Malaval C., Gille A., Kero J., Offermanns S. (2011). Nicotinic acid inhibits progression of atherosclerosis in mice through its receptor GPR109A expressed by immune cells. Journal of Clinical Investigation.

[96] Hegyi J., Schwartz R.A., Hegyi V. (2004). Pellagra: dermatitis, dementia, and diarrhea. International Journal of Dermatology.

[97] Singh N., Gurav A., Sivaprakasam S., Brady E., Padia R., Shi H. (2014). Activation of Gpr109a, receptor for niacin and the commensal metabolite butyrate, suppresses colonic inflammation and carcinogenesis. Immunity.

[98] Rebeck G.W. (2004). Cholesterol efflux as a critical component of Alzheimer's disease pathogenesis. Journal of Molecular Neuroscience.

[99] Riddell D.R., Zhou H., Comery T.A., Kouranova E., Lo C.F., Warwick H.K. (2007). The LXR agonist TO901317 selectively lowers hippocampal Abeta42 and improves memory in the Tg2576 mouse model of Alzheimer's disease. Molecular and Cellular Neuroscience.

[100] Zhao S.P., Yang J., Li J., Dong S.Z., Wu Z.H. (2008). Effect of niacin on LXRalpha and PPARgamma expression and HDL-induced cholesterol efflux in adipocytes of hypercholesterolemic rabbits. International Journal of Cardiology.

[101] Ljungberg M.C., Ali Y.O., Zhu J., Wu C.-S., Oka K., Zhai R.G. (2011). CREB-activity and nmnat2 transcription are down-regulated prior to neurodegeneration, while NMNAT2 over-expression is neuroprotective, in a mouse model of human tauopathy. Human Molecular Genetics.

[102] Green K.N., Steffan J.S., Martinez-Coria H., Sun X., Schreiber S.S., Thompson L.M. (2008). Nicotinamide restores cognition in Alzheimer's disease transgenic mice via a mechanism involving sirtuin inhibition and selective reduction of Thr231-phosphotau. Journal of Neuroscience : The Official Journal of the Society for Neuroscience.

[103] Ali Y.O., Allen H.M., Yu L., Li-Kroeger D., Bakhshizadehmahmoudi D., Hatcher A. (2016). NMNAT2:HSP90 complex mediates proteostasis in proteinopathies. PLoS Biology.

[104] Bayrakdar E.T., Armagan G., Uyanikgil Y., Kanit L., Koylu E., Yalcin A. (2014). Ex vivo protective effects of nicotinamide and 3-aminobenzamide on rat synaptosomes treated with Aβ(1-42). Cell Biochemistry and Function.

[105] Young G.S., Jacobson E.L., Kirkland J.B. (2007). Water maze performance in young male Long-Evans rats is inversely affected by dietary intakes of niacin and may be linked to levels of the NAD+ metabolite cADPR. Journal of Nutrition.

[106] Blacher E., Dadali T., Bespalko A., Haupenthal V., Grimm M., Hartmann T. (2015). Alzheimer's disease pathology is attenuated in a CD38-deficient mouse model: CD38 and AD Pathology. Annals of Neurology.

[107] Alexander G.E. (2004). Biology of Parkinson's disease: pathogenesis and pathophysiology of a multisystem neurodegenerative disorder. Dialogues in Clinical Neuroscience.

[108] DeMaagd G., Philip A. (2015). *Parkinson's Disease and its management: Part 1: disease Entity, risk factors, pathophysiology, clinical presentation, and diagnosis.* P & T : a peer-reviewed. Journal for Formulary Management.

[109] Helmich R.C., Hallett M., Deuschl G., Toni I., Bloem B.R. (2012). Cerebral causes and consequences of parkinsonian resting tremor: a tale of two circuits?. Brain : A Journal of Neurology.

[110] Albin R.L., Young A.B., Penney J.B. (1989). The functional anatomy of basal ganglia disorders. Trends in Neurosciences.

[111] Wakade C., Chong R., Bradley E., Thomas B., Morgan J. (2014). Upregulation of GPR109A in Parkinson's disease. PloS One.

[112] Wakade C., Chong R., Bradley E., Morgan J.C. (2015). Low-dose niacin supplementation modulates GPR109A, niacin index and ameliorates Parkinson's disease symptoms without side effects. Clinical Case Reports.

[113] Alisky J.M. (2005). Niacin improved rigidity and bradykinesia in a Parkinson's disease patient but also caused unacceptable nightmares and skin rash--a case report. Nutritional Neuroscience.

[114] Gadol E., Mestayer R., Grant R., Grigoryev Y., Gibson S., Happel M. (2019). A case of Parkinson's disease symptom reduction with intravenous NAD +.

[115] Williams A., Ramsden D. (2005). Nicotinamide: a double edged sword. Parkinsonism & Related Disorders.

[116] Lehmann S., Costa A.C., Celardo I., Loh S.H.Y., Martins L.M. (2016). Parp mutations protect against mitochondrial dysfunction and neurodegeneration in a PARKIN model of Parkinson's disease. Cell Death & Disease.

[117] Lehmann S., Loh S.H., Martins L.M. (2017). Enhancing NAD(+) salvage metabolism is neuroprotective in a PINK1 model of Parkinson's disease. Biol Open.

[118] Schöndorf D.C., Ivanyuk D., Baden P., Sanchez-Martinez A., De Cicco S., Yu C. (2018). The NAD+ precursor nicotinamide riboside rescues mitochondrial defects and neuronal loss in iPSC and fly models of Parkinson's disease. Cell Reports.

[119] Singh P., Hanson P.S., Morris C.M. (2017). SIRT1 ameliorates oxidative stress induced neural cell death and is down-regulated in Parkinson's disease. BMC Neuroscience.

[120] Jeong H., Cohen D.E., Cui L., Supinski A., Savas J.N., Mazzulli J.R. (2011). Sirt1 mediates neuroprotection from mutant huntingtin by activation of the TORC1 and CREB transcriptional pathway. Nature Medicine.

[121] Beal M.F., Matson W.R., Swartz K.J., Gamache P.H., Bird E.D. (1990). Kynurenine pathway measurements in Huntington's disease striatum: evidence for reduced formation of kynurenic acid. Journal of Neurochemistry.

[122] Zwilling D., Huang S.-Y., Sathyasaikumar Korrapati V., Notarangelo Francesca M., Guidetti P., Wu H.-Q. (2011). Kynurenine 3-monooxygenase inhibition in blood ameliorates neurodegeneration. Cell.

[123] Lloret A., Beal M.F. (2019). PGC-1α, sirtuins and PARPs in Huntington's disease and other neurodegenerative conditions: NAD+ to rule them all. Neurochemical Research.

[124] Pallos J., Bodai L., Lukacsovich T., Purcell J.M., Steffan J.S., Thompson L.M. (2008). Inhibition of specific HDACs and sirtuins suppresses pathogenesis in a Drosophila model of Huntington's disease. Human Molecular Genetics.

[125] Paldino E., Cardinale A., D'Angelo V., Sauve I., Giampà C., Fusco F.R. (2017). Selective sparing of striatal interneurons after poly (ADP-Ribose) polymerase 1 inhibition in the R6/2 mouse model of Huntington's disease. Frontiers in Neuroanatomy.

[126] O'Neill S., O'Driscoll L. (2015). Metabolic syndrome: a closer look at the growing epidemic and its associated pathologies. Obesity Reviews.

[127] Okabe K., Yaku K., Tobe K., Nakagawa T. (2019). Implications of altered NAD metabolism in metabolic disorders. Journal of Biomedical Science.

[128] Garten A., Schuster S., Penke M., Gorski T., de Giorgis T., Kiess W. (2015). Physiological and pathophysiological roles of NAMPT and NAD metabolism. Nature Reviews Endocrinology.

[129] Wu J., Jin Z., Zheng H., Yan L.J. (2016). Sources and implications of NADH/NAD(+) redox imbalance in diabetes and its complications. Diabetes, Metabolic Syndrome and Obesity: Targets and Therapy.

[130] Zhou S., Tang X., Chen H.-Z. (2018). Sirtuins and insulin resistance. Frontiers in Endocrinology.

[131] Revollo J.R., Körner A., Mills K.F., Satoh A., Wang T., Garten A. (2007). Nampt/PBEF/Visfatin regulates insulin secretion in beta cells as a systemic NAD biosynthetic enzyme. Cell Metabolism.

[132] Trammell S.A.J., Weidemann B.J., Chadda A., Yorek M.S., Holmes A., Coppey L.J. (2016). Nicotinamide riboside opposes type 2 diabetes and neuropathy in mice. Scientific Reports.

[133] Fan L., Cacicedo J.M., Ido Y. (2020). Impaired nicotinamide adenine dinucleotide (NAD+) metabolism in diabetes and diabetic tissues: implications for nicotinamide-related compound treatment. Journal of Diabetes Investigation.

[134] Zhou S.S., Li D., Sun W.P., Guo M., Lun Y.Z., Zhou Y.M. (2009). Nicotinamide overload may play a role in the development of type 2 diabetes. World Journal of Gastroenterology.

[135] Hou W.-L., Yin J., Alimujiang M., Yu X.-Y., Ai L.-G., Bao Y.-Q. (2018). Inhibition of mitochondrial complex I improves glucose metabolism independently of AMPK activation. Journal of Cellular and Molecular Medicine.

[136] Dollerup O.L., Christensen B., Svart M., Schmidt M.S., Sulek K., Ringgaard S. (2018). A randomized placebo-controlled clinical trial of nicotinamide riboside in obese men: safety, insulin-sensitivity, and lipid-mobilizing effects. American Journal of Clinical Nutrition.

[137] Jokinen R., Pirnes-Karhu S., Pietiläinen K.H., Pirinen E. (2017). Adipose tissue NAD+-homeostasis, sirtuins and poly(ADP-ribose) polymerases -important players in mitochondrial metabolism and metabolic health. Redox Biology.

[138] Bournat J.C., Brown C.W. (2010). Mitochondrial dysfunction in obesity. Current Opinion in Endocrinology, Diabetes, and Obesity.

[139] Gaddipati R., Sasikala M., Padaki N., Mukherjee R.M., Sekaran A., Jayaraj-Mansard M. (2010). Visceral adipose tissue visfatin in non-alcoholic fatty liver disease. Annals of Hepatology.

[140] Yoon M.J., Yoshida M., Johnson S., Takikawa A., Usui I., Tobe K. (2015). SIRT1-Mediated eNAMPT secretion from adipose tissue regulates hypothalamic NAD+ and function in mice. Cell Metabolism.

[141] Yoshida M., Satoh A., Lin J.B., Mills K.F., Sasaki Y., Rensing N. (2019). Extracellular vesicle-contained eNAMPT delays aging and extends lifespan in mice. Cell Metabolism.

[142] Nielsen K.N., Peics J., Ma T., Karavaeva I., Dall M., Chubanava S. (2018). NAMPT-mediated NAD(+) biosynthesis is indispensable for adipose tissue plasticity and development of obesity. Molecular Metabolism.

[143] Dall M., Trammell S.A.J., Asping M., Hassing A.S., Agerholm M., Vienberg S.G. (2019). Mitochondrial function in liver cells is resistant to perturbations in NAD+ salvage capacity. Journal of Biological Chemistry.

[144] Guarente L. (2006). Sirtuins as potential targets for metabolic syndrome. Nature.

[145] Pfluger P.T., Herranz D., Velasco-Miguel S., Serrano M., Tschöp M.H. (2008). Sirt1 protects against high-fat diet-induced metabolic damage. Proceedings of the National Academy of Sciences of the United States of America.

[146] Lagouge M., Argmann C., Gerhart-Hines Z., Meziane H., Lerin C., Daussin F. (2006). Resveratrol improves mitochondrial function and protects against metabolic disease by activating SIRT1 and PGC-1alpha. Cell.

[147] Cummins T.D., Holden C.R., Sansbury B.E., Gibb A.A., Shah J., Zafar N. (2014). Metabolic remodeling of white adipose tissue in obesity. American Journal of Physiology Endocrinology and Metabolism.

[148] Drew J.E., Farquharson A.J., Horgan G.W., Williams L.M. (2016). Tissue-specific regulation of sirtuin and nicotinamide adenine dinucleotide biosynthetic pathways identified in C57Bl/6 mice in response to high-fat feeding. The Journal of Nutritional Biochemistry.

[149] Chalkiadaki A., Guarente L. (2012). High-fat diet triggers inflammation-induced cleavage of SIRT1 in adipose tissue to promote metabolic dysfunction. Cell Metabolism.

[150] Wang F., Tong Q. (2009). SIRT2 suppresses adipocyte differentiation by deacetylating FOXO1 and enhancing FOXO1's repressive interaction with PPARgamma. Molecular Biology of the Cell.

[151] Cohen H.Y., Miller C., Bitterman K.J., Wall N.R., Hekking B., Kessler B. (2004). Calorie restriction promotes mammalian cell survival by inducing the SIRT1 deacetylase. Science.

[152] Chen D., Bruno J., Easlon E., Lin S.J., Cheng H.L., Alt F.W. (2008). Tissue-specific regulation of SIRT1 by calorie restriction. Genes & Development.

[153] Laurent G., German N.J., Saha A.K., de Boer V.C.J., Davies M., Koves T.R. (2013). SIRT4 coordinates the balance between lipid synthesis and catabolism by repressing malonyl CoA decarboxylase. Molecular Cell.

[154] Satapati S., Kucejova B., Duarte J.A., Fletcher J.A., Reynolds L., Sunny N.E. (2015). Mitochondrial metabolism mediates oxidative stress and inflammation in fatty liver. Journal of Clinical Investigation.

[155] Dahl T.B., Haukeland J.W., Yndestad A., Ranheim T., Gladhaug I.P., Damås J.K. (2010). Intracellular nicotinamide phosphoribosyltransferase protects against hepatocyte apoptosis and is down-regulated in non-alcoholic fatty liver disease. Journal of Clinical Endocrinology & Metabolism.

[156] Wu T., Liu Y.H., Fu Y.C., Liu X.M., Zhou X.H. (2014). Direct evidence of sirtuin downregulation in the liver of non-alcoholic fatty liver disease patients. Annals of Clinical Laboratory Science.

[157] Amano H., Chaudhury A., Rodriguez-Aguayo C., Lu L., Akhanov V., Catic A. (2019). Telomere dysfunction induces sirtuin repression that drives telomere-dependent disease. Cell Metabolism.

[158] Gariani K., Menzies K.J., Ryu D., Wegner C.J., Wang X., Ropelle E.R. (2016). Eliciting the mitochondrial unfolded protein response by nicotinamide adenine dinucleotide repletion reverses fatty liver disease in mice. Hepatology.

[159] Li Y., Wong K., Giles A., Jiang J., Lee J.W., Adams A.C. (2014). Hepatic SIRT1 attenuates hepatic steatosis and controls energy balance in mice by inducing fibroblast growth factor 21. Gastroenterology.

[160] Shen C., Dou X., Ma Y., Ma W., Li S., Song Z. (2017). Nicotinamide protects hepatocytes against palmitate-induced lipotoxicity via SIRT1-dependent autophagy induction. Nutrition Research.

[161] Ralto K.M., Rhee E.P., Parikh S.M. (2020). NAD+ homeostasis in renal health and disease. Nature Reviews Nephrology.

[162] Zhuo L., Fu B., Bai X., Zhang B., Wu L., Cui J. (2011). NAD blocks high glucose induced mesangial hypertrophy via activation of the sirtuins-AMPK-mTOR pathway. Cellular Physiology and Biochemistry.

[163] Guan Y., Wang S.-R., Huang X.-Z., Xie Q.-H., Xu Y.-Y., Shang D. (2017). Nicotinamide mononucleotide, an NAD(+) precursor, rescues age-associated susceptibility to AKI in a sirtuin 1-dependent manner. Journal of the American Society of Nephrology : JASN.

[164] Tran M.T., Zsengeller Z.K., Berg A.H., Khankin E.V., Bhasin M.K., Kim W. (2016). PGC1α drives NAD biosynthesis linking oxidative metabolism to renal protection. Nature.

[165] Oh G.-S., Kim H.-J., Choi J.-H., Shen A., Choe S.-K., Karna A. (2014). Pharmacological activation of NQO1 increases NAD⁺ levels and attenuates cisplatin-mediated acute kidney injury in mice. Kidney International.

[166] Poyan Mehr A., Tran M.T., Ralto K.M., Leaf D.E., Washco V., Messmer J. (2018). De novo NAD(+) biosynthetic impairment in acute kidney injury in humans. Nature Medicine.

[167] Jose C., Bellance N., Rossignol R. (2011). Choosing between glycolysis and oxidative phosphorylation: a tumor's dilemma?. Biochimica et Biophysica Acta.

[168] Fadaka A., Ajiboye B., Ojo O., Adewale O., Olayide I., Emuowhochere R. (2017). Biology of glucose metabolization in cancer cells. Journal of Oncological Sciences.

[169] Ashton T.M., McKenna W.G., Kunz-Schughart L.A., Higgins G.S. (2018). Oxidative phosphorylation as an emerging target in cancer therapy. Clinical Cancer Research.

[170] Yaku K., Okabe K., Hikosaka K., Nakagawa T. (2018). NAD metabolism in cancer therapeutics. Frontiers in oncology.

[171] Shackelford R.E., Mayhall K., Maxwell N.M., Kandil E., Coppola D. (2013). Nicotinamide phosphoribosyltransferase in malignancy: a review. Genes & Cancer.

[172] Menssen A., Hydbring P., Kapelle K., Vervoorts J., Diebold J., Lüscher B. (2012). The c-MYC oncoprotein, the NAMPT enzyme, the SIRT1-inhibitor DBC1, and the SIRT1 deacetylase form a positive feedback loop. Proceedings of the National Academy of Sciences of the United States of America.

[173] Tarrado-Castellarnau M., de Atauri P., Cascante M. (2016). Oncogenic regulation of tumor metabolic reprogramming. Oncotarget.

[174] Grolla A.A., Torretta S., Gnemmi I., Amoruso A., Orsomando G., Gatti M. (2015). Nicotinamide phosphoribosyltransferase (NAMPT/PBEF/visfatin) is a tumoural cytokine released from melanoma. Pigment Cell & Melanoma Research.

[175] Audrito V., Managò A., Zamporlini F., Rulli E., Gaudino F., Madonna G. (2018). Extracellular nicotinamide phosphoribosyltransferase (eNAMPT) is a novel marker for patients with BRAF-mutated metastatic melanoma. Oncotarget.

[176] Audrito V., Serra S., Brusa D., Mazzola F., Arruga F., Vaisitti T. (2015). Extracellular nicotinamide phosphoribosyltransferase (NAMPT) promotes M2 macrophage polarization in chronic lymphocytic leukemia. Blood.

[177] Sun Y., Zhu S., Wu Z., Huang Y., Liu C., Tang S. (2017). Elevated serum visfatin levels are associated with poor prognosis of hepatocellular carcinoma. Oncotarget.

[178] Soncini D., Caffa I., Zoppoli G., Cea M., Cagnetta A., Passalacqua M. (2014). Nicotinamide phosphoribosyltransferase promotes epithelial-to-mesenchymal transition as a soluble factor independent of its enzymatic activity. Journal of Biological Chemistry.

[179] Tummala K.S., Gomes A.L., Yilmaz M., Graña O., Bakiri L., Ruppen I. (2014). Inhibition of de novo NAD(+) synthesis by oncogenic URI causes liver tumorigenesis through DNA damage. Cancer Cell.

[180] Watson M., Roulston A., Bélec L., Billot X., Marcellus R., Bédard D. (2009). The small molecule GMX1778 is a potent inhibitor of NAD+ biosynthesis: strategy for enhanced therapy in nicotinic acid phosphoribosyltransferase 1-deficient tumors. Molecular and Cellular Biology.

[181] Tateishi K., Wakimoto H., Iafrate A.J., Tanaka S., Loebel F., Lelic N. (2015). Extreme vulnerability of IDH1 mutant cancers to NAD+ depletion. Cancer Cell.

[182] Piacente F., Caffa I., Ravera S., Sociali G., Passalacqua M., Vellone V.G. (2017). Nicotinic acid phosphoribosyltransferase regulates cancer cell metabolism, susceptibility to NAMPT inhibitors, and DNA repair. Cancer Research.

[183] Murata M.M., Kong X., Moncada E., Chen Y., Imamura H., Wang P. (2019). NAD+ consumption by PARP1 in response to DNA damage triggers metabolic shift critical for damaged cell survival. Molecular Biology of the Cell.

[184] Hurtado-Bagès S., Knobloch G., Ladurner A.G., Buschbeck M. (2020). The taming of PARP1 and its impact on NAD+ metabolism. Molecular Metabolism.

[185] Huang X., Motea E.A., Moore Z.R., Yao J., Dong Y., Chakrabarti G. (2016). Leveraging an NQO1 bioactivatable drug for tumor-selective use of poly(ADP-ribose) polymerase inhibitors. Cancer Cell.

[186] Zhang T., Berrocal J.G., Frizzell K.M., Gamble M.J., DuMond M.E., Krishnakumar R. (2009). Enzymes in the NAD+ salvage pathway regulate SIRT1 activity at target gene promoters. Journal of Biological Chemistry.

[187] van der Veer E., Ho C., O'Neil C., Barbosa N., Scott R., Cregan S.P. (2007). Extension of human cell lifespan by nicotinamide phosphoribosyltransferase. Journal of Biological Chemistry.

[188] Lucena-Cacace A., Otero-Albiol D., Jiménez-García M.P., Peinado-Serrano J., Carnero A. (2017). NAMPT overexpression induces cancer stemness and defines a novel tumor signature for glioma prognosis. Oncotarget.

[189] Wang B., Hasan M.K., Alvarado E., Yuan H., Wu H., Chen W.Y. (2011). NAMPT overexpression in prostate cancer and its contribution to tumor cell survival and stress response. Oncogene.

[190] Lucena-Cacace A., Otero-Albiol D., Jiménez-García M.P., Muñoz-Galvan S., Carnero A. (2018). NAMPT is a potent oncogene in colon cancer progression that modulates cancer stem cell properties and resistance to therapy through Sirt1 and PARP. Clinical Cancer Research.

[191] Son M.J., Ryu J.S., Kim J.Y., Kwon Y., Chung K.S., Mun S.J. (2017). Upregulation of mitochondrial NAD(+) levels impairs the clonogenicity of SSEA1(+) glioblastoma tumor-initiating cells. Experimental & Molecular Medicine.

[192] Ren T., Zhang H., Wang J., Zhu J., Jin M., Wu Y. (2017). MCU-dependent mitochondrial Ca(2+) inhibits NAD(+)/SIRT3/SOD2 pathway to promote ROS production and metastasis of HCC cells. Oncogene.

[193] Li Y., Zhang M., Dorfman R.G., Pan Y., Tang D., Xu L. (2018). SIRT2 promotes the migration and invasion of gastric cancer through RAS/ERK/JNK/MMP-9 pathway by increasing PEPCK1-related metabolism. Neoplasia (New York, N.Y.).

[194] Chen J., Chan A.W., To K.F., Chen W., Zhang Z., Ren J. (2013). SIRT2 overexpression in hepatocellular carcinoma mediates epithelial to mesenchymal transition by protein kinase B/glycogen synthase kinase-3β/β-catenin signaling. Hepatology.

[195] Xu S.-N., Wang T.-S., Li X., Wang Y.-P. (2016). SIRT2 activates G6PD to enhance NADPH production and promote leukaemia cell proliferation. Scientific Reports.

[196] Smith J.S. (2002). Human Sir2 and the ‘silencing’ of p53 activity. Trends in Cell Biology.

[197] Tucci, P., Caloric restriction: is mammalian life extension linked to p53? Aging. 4(8): p. 525-534. 10.18632/aging.100481 PMC3461340 22983298

[198] Hwang E.S., Song S.B. (2020). Possible adverse effects of high-dose nicotinamide: mechanisms and safety assessment. Biomolecules.

[199] Jacobson T.A. (2010). A "hot" topic in dyslipidemia management--"how to beat a flush": optimizing niacin tolerability to promote long-term treatment adherence and coronary disease prevention. Mayo Clinic Proceedings.

[200] Wang X., Zhang Q., Bao R., Zhang N., Wang Y., Polo-Parada L. (2017). Deletion of nampt in projection neurons of adult mice leads to motor dysfunction, neurodegeneration, and death. Cell Reports.

[201] Stone T.W., Darlington L.G. (2013). The kynurenine pathway as a therapeutic target in cognitive and neurodegenerative disorders. British Journal of Pharmacology.

[202] Yan L.-J. (2018). Redox imbalance stress in diabetes mellitus: role of the polyol pathway. Animal Models and Experimental Medicine.

[203] Yan L.-J. (2014). Pathogenesis of chronic hyperglycemia: from reductive stress to oxidative stress. Journal of Diabetes Research.

[204] Chiarugi A., Dölle C., Felici R., Ziegler M. (2012). The NAD metabolome — a key determinant of cancer cell biology. Nature Reviews Cancer.

